# Research on Optimal Convergence Design of Low Intercept Point-Like Beam for FDA-MIMO Radio Detector Based on Beam Entropy

**DOI:** 10.3390/e27040421

**Published:** 2025-04-12

**Authors:** Jinwei Jia, Min Gao, Yuying Liang, Xinyu Dao, Yuanwei Yin, Zhuangzhi Han

**Affiliations:** 1 Shijiazhuang Campus, Army Engineering University, Shijiazhuang 050003, China; serverus@aeu.edu.cn (J.J.); albus2022@163.com (M.G.); potter2022@126.com (Y.Y.); 2 School of Aerospace Engineering, Nanchang Institute of Technology, Nanchang 330000, China; liangyy@nut.edu.cn; 3 Beijing Special Electromechanical Research Institute, Beijing 100020, China; daoxy001@126.com

**Keywords:** FDA-MIMO, radio detector, low probability of intercept, optimal convergence of point-like, beam entropy

## Abstract

The technology of anti-informational interference is a research hotspot in radio detectors. According to the workflow of first interception and then interference for the jammer, improving low interception can fundamentally improve the anti-jamming ability of the radio detector. Airspace low interception is one of the most promising research directions. FDA-MIMO technology holds significant potential for application in this field. Therefore, this paper investigates the design principle of an FDA-MIMO radio detector with low beam entropy. From the perspectives of information acquisition and countermeasure, the spatial low interception of a radio detector is defined by beam entropy. In this paper, the power peak point and drop point are set in a relatively close range (Δr), ensuring the rapid attenuation of beam amplitude over short distances. Consequently, the design principle of the FDA-MIMO low interception point beam based on the array frequency offset setting formula is obtained, and the optimal beam convergence is realized. Simulation results show that the half-power beam widths of FDA-MIMO point-like beams are 1 m in the distance dimension and 9 degrees in the beamwidth dimension, with a beam entropy of 11. Compared with other classical frequency offset setting methods, the proposed method demonstrates significantly superior beam performance, particularly in terms of low intercept characteristics. The design principle proposed in this paper provides theoretical support for the low intercept beam design of the FDA-MIMO radio detector, thereby reducing the probability of jammers acquiring signal parameters and enhancing both the low intercept performance and anti-jamming capabilities of the radio detector.

## 1. Introduction

The detector plays a crucial role in the detection and control of terminal damage within weapon systems, directly influencing the effectiveness of the weapon system [1]. The radio detector, characterized by its simple structure, superior precision in ranging, and capability to effectively engage both aerial and ground targets, has been widely used at the present stage [2]. In the modern battlefield, the electromagnetic environment is becoming increasingly complex, posing significant challenges for radio detectors to contend with many electromagnetic interferences. In particular, the repeater deception jamming, which is implemented by the fourth-generation jammer based on the digital radio frequency memory (DRFM) technology, can easily make the radio detector lose efficacy. Therefore, it is imperative to investigate and enhance the resistance of radio detectors against such jamming techniques, thereby improving their capability to withstand information interference.

In recent years, scholars have conducted extensive research on the anti-information interference of the radio detector [3,4,5,6], which can be categorized into multi-mode anti-interference based on information fusion and single-mode anti-interference based on the radio signal. The multi-mode anti-interference approach primarily integrates radio detection with other proximity detection technologies and uses multiple sensors to analyze signals in different domains. The detonation signal can only be output when the target signals generated by different sensors achieve a match. Currently, the research mainly focuses on the multi-mode radio detector of “radio signals + laser signals” [7,8,9,10,11]. The literature [10,11] proposed a composite detection technology combining laser and radio along with the initiation criterion for radio detectors. This approach effectively solved the problems of laser detection technology in resisting interference from clouds, smoke, rain and snow, and radio-transmitted deceptive jamming. It significantly improved the signal-to-noise ratio. Moreover, a prototype was established, and the effectiveness of the composite detection technology in anti-interference was validated. However, in engineering practice, the multi-mode radio detector necessitates the design of transceiver modules that support more than two distinct signal systems, which require a relatively large space. The constrained space within the radio detector has emerged as a critical limiting factor for advancing multi-mode radio detectors.

The single-mode anti-interference based on radio signal is generally divided into three directions: **signal parameter modulation anti-interference, ultra-wideband anti-interference, and low probability intercept anti-jamming.** Regarding signal parameter modulation anti-interference direction, the literature [12] proposed an anti-interference method for the pulse Doppler radio detector based on M-sequence pseudo-code phase modulation. The response expressions at all levels during the detection process of the radio detector were theoretically derived, and the anti-interference performance of the radio detector was quantified. However, calculating the height of the radio detector requires the use of pseudo-code sequence autocorrelation. Jammers can directly copy and forward signals without mastering the specific system (form) of radio detector signals. Therefore, it is challenging for the pseudo-code phase modulation method to counter the spoofing interference based on DRFM. The literature [13,14,15,16] introduced a frequency-hopping signal where the frequency points were modulated by a pseudo-random sequence within a specified range, resulting in a random frequency pattern. Nevertheless, the jammer based on DRFM can also detect the frequency of signals at a relatively fast speed. Moreover, the frequency-hopping range of the radio detector is limited and it is easy to be detected by the jammer. Consequently, using signal parameter hopping alone presents challenges in effectively countering transmitted deceptive jamming.

In the field of ultra-wideband anti-jamming, Yan et al. [17] studied the anti-interference performance of ultra-wideband radio detectors. The signal-to-stem ratio gain of correlation reception in the presence of Gaussian white noise and sinusoidal interference was derived in detail, and the signal characteristic parameters that affect the anti-jamming ability of the UWB radio detector were obtained. The quantitative analysis and simulation results show that the UWB radio detector had good anti-jamming performance against noise and sine interference. However, due to the reliance on correlation detection for distance determination, UWB radio detectors exhibited relatively limited resistance to forwarding spoofing interference. Zhu et al. [18] studied the intelligent power distribution method of carrier-free UWB radio detectors in different frequency bands to achieve low interception. However, given the wide operating frequency band of UWB detectors, they are susceptible to unintentional interference from any frequency point within the band. Therefore, while these detectors demonstrate superior anti-reconnaissance performance, their overall anti-interference capability remains relatively weak [19].

The third direction is low interception anti-interference. Given that the jammer first intercepts the signal, the low interception radio detector has significant anti-jamming potential. The low intercept radio detector encompasses three research directions: signal energy detection low intercept, parameter measurement low intercept, and spatial low intercept. According to the literature [20,21,22], when the radio frequency signal approaches the resonance frequencies of water molecules and oxygen molecules in the atmosphere, the energy of the radio frequency signal will be absorbed, thereby reducing its detectability. By selecting the detection signal frequency of the radio detector in the frequency band with greater signal attenuation, the low probability of intercept performance of the radio detector can be improved. It should be noted that, with the rapid development of radio frequency components, modern jammers are now equipped with high-sensitivity receiving systems. In the presence of a sensitivity jammer, it is challenging for radio detectors to achieve low interception of detector signal energy detection. At the same time, the jammer can measure the frequency and other parameters of the radio detector signal quickly and with high precision. Therefore, it is difficult for radio detectors to achieve low interception when measuring signal parameters. This paper addresses this issue by focusing on airspace low intercept techniques and employing advanced signal design technologies to overcome the associated difficulties. Consequently, the low intercept and anti-jamming objectives for radio detectors are realized.

According to the above analysis, the radio detector has a broad application prospect in airspace low interception. From the perspective of information theory, the low interception radio detector ensures that the adversary acquires minimal information from its signal and beam, that is, the low entropy radio detector. Therefore, the concept of beam entropy is proposed in this paper to guide the design of a low intercept radio detector. Based on the characteristics of an S-shaped beam pattern in the frequency diverse array (FDA), we investigate the design of a low intercept spot-shaped beam from the perspective of FDA. By rapidly attenuating the amplitude of the wave crest in a small area (Δr), the frequency offset setting formula of each array element is derived using the multiple-input multiple-output (MIMO) beam pattern function. The design principle of a low interception point beam based on the formula of array element frequency offset is obtained. The FDA-MIMO array can synthesize energy-concentrated spot beams in space, significantly improving the spatial low intercept performance of radio short-range detectors. Furthermore, the principle of point-beam design proposed in this paper provides theoretical support for point-beam design and guides the development of low intercept beam designs for radio detectors based on FDA-MIMO technology, thereby reducing the likelihood of interception by jammer reconnaissance.

**Figure 1:**
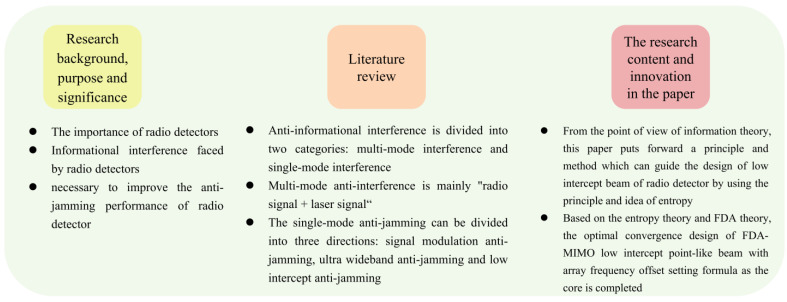
The logic diagram of the introduction.

The logic diagram for writing the introduction of this paper is shown in Figure 1. The contributions of this study are as follows.

1. From the point of view of information theory, this paper puts forward a principle and method that can guide the design of a low intercept beam of the radio detector using the principle and idea of entropy.
2. Furthermore, using entropy theory and FDA theory, the optimal convergence design of the FDA-MIMO low intercept point-like beam with an array frequency offset setting formula as the core is completed.

It should be noted that this paper does not focus on the anti-interference capabilities of radio detectors. Instead, it aims to design low intercept beams for radio detectors to minimize the enemy’s acquisition of our signal parameter information. The simulation results of the proposed method in an ideal scenario are shown in Section 5. When considering the real-world operating conditions of radio detectors, noise and interference can occur during beam propagation. This results in the enemy receiving a weaker signal, thereby obtaining less signal information from us. Such a situation is more conducive to achieving our objective of low interception and reduces the probability of enemy detection.

This paper is organized as follows. Section 2 presents the evaluation parameter design of low intercept performance based on entropy theory. Section 3 introduces the basic theory of FDA. Section 4 derives the formula for the design principle of a low intercept point-like beam. Section 5 provides simulations and discussions on the signal design principle proposed in this paper. Section 6 is the conclusion.

## 2. Evaluation Parameter Design of Low Intercept Performance Based on Entropy Theory

The evaluation parameters of low intercept performance are crucial for guiding the design of radio detectors. Numerous evaluation indexes of low intercept of radio detectors have been proposed, but no consensus has been reached. Rhee et al. [23] proposed evaluation indexes such as frequency entropy, frequency level, and frequency hopping. Wu et al. [24] selected parameters (e.g., broadband width product, signal complexity, peak power, minimum dwell time, sidelobe size, and beamwidth) and used the improved G-GIFSS algorithm to evaluate the low interception performance of the radio detector. Despite their comprehensiveness, these methods require extensive parameter calculations and thus fail to meet the requirement for concise computation. Chen et al. [25] proposed that the transmitting power of the signal can serve as an evaluation parameter for low intercept probability. They argued that the lower the transmitting power correlates with a reduced likelihood of interception. However, this method is limited and may introduce subjectivity, making it challenging to objectively and comprehensively assess the low intercept performance of radio detectors. Therefore, establishing a simple, objective, and fair evaluation index of the low intercept performance is crucial, effectively guiding the design of the low intercept beam.

Interception refers to the detection of radio signals by enemy jammers, which allows the adversary to acquire our information. A low interception beam minimizes the amount of information that can be obtained by the enemy. Jia and Dao from the Army Engineering University proposed approximate entropy to measure the complexity of chaotic sequences. Approximate entropy is used to evaluate the jump performance of signal parameters modulated by chaotic sequence [14,15]. Subsequently, the low interception performance of the signal is evaluated. However, this method evaluates approximate entropy solely based on signal parameters and does not account for the convergence of the beam in space or the low interception performance in the beam’s airspace.

Therefore, from the perspective of information acquisition and confrontation between radio detectors and intercepting receivers, this paper defines beam entropy to quantitatively evaluate the low intercept performance of the beam. Entropy is a fundamental parameter in thermodynamics that characterizes the state of matter, with its physical meaning quantifying the degree of disorder within a system. Information entropy is a basic concept of information theory, which describes the uncertainty of various possible events occurring in an information source. Information entropy was first proposed by Shannon [26].

Information entropy addresses the problem of quantitatively measuring information. The formula for calculating information entropy is as follows:

(1) $$H=-\sum_{i=1}^{n}p_{i}\log p_{i}.$$

where $p_{i}$ represents the probability of event *i* occurring in the event set; *n* indicates the total number of events in the event set. The logarithmic function is based on 2. In this paper, beam entropy is defined to quantitatively evaluate the low intercept performance of a beam. The beam entropy can be expressed by Equation (2):

(2) $$E_{b}=-\int_{-\frac{\pi }{2}}^{\frac{\pi }{2}}\int_{0}^{R}p(r,\theta )\ln p(r,\theta )\;drd\theta$$

where *r* is the beam detection distance at any point $\left(r,\theta \right)$ within the beam detection range; θ is the beam detection angle at any point $\left(r,\theta \right)$ within the beam detection range; $p(r,\theta )=\frac{\left|A(r,\theta )\right|}{\sum_{\theta =-\pi /2}^{\pi /2}\sum_{r=0}^{R}\left|A(r,\theta )\right|}$. $A\left(r,\theta \right)$ represents the beam amplitude of a certain $\left(r,\theta \right)$ in the beam detection range. The unit of $E_{b}$ is bits. In theory, r and θ are beam parameters and should be continuous parameters. However, radio signal processing equipment is mainly digital in engineering practice. Consequently, beam entropy is primarily achieved through the digitization and discretization of parameters. Therefore, Equation (2) in engineering practice can be rewritten as:

(3) $$E_{b}=-\sum_{\theta =-\pi /2}^{\pi /2}\sum_{r=0}^{R}p(r,\theta )\ln p(r,\theta )$$

Beam entropy $E_{b}$ is used to reflect the average information content of the beam. A lower beam entropy value indicates greater beam convergence, which enhances the low intercept performance of the beam. In the existing spatial beamforming technology, FDA-MIMO technology can form a narrow beam in both distance and beamwidth, resulting in lower beam entropy. Therefore, this paper employs FDA-MIMO technology to design a low intercept radio detector.

## 3. Basic Theory of FDA-MIMO

The concept of FDA was first proposed in the literature [27,28]. FDA introduces a frequency offset to each coherent signal’s center frequency, where the offset is significantly smaller than the center frequency itself. Although the center frequency of each signal radiated out is offset, the main frequency components remain overlapping. This approach differs from orthogonal frequency division multiplexing [29] (OFDM) and MIMO required for frequency orthogonality. The physical characteristics of FDA are similar to those of phased arrays [30].

Due to the unique S-shaped beam pattern of FDA, the concept of frequency array has attracted wide attention [31,32]. There are a large number of studies on FDA.

**Figure 2:**
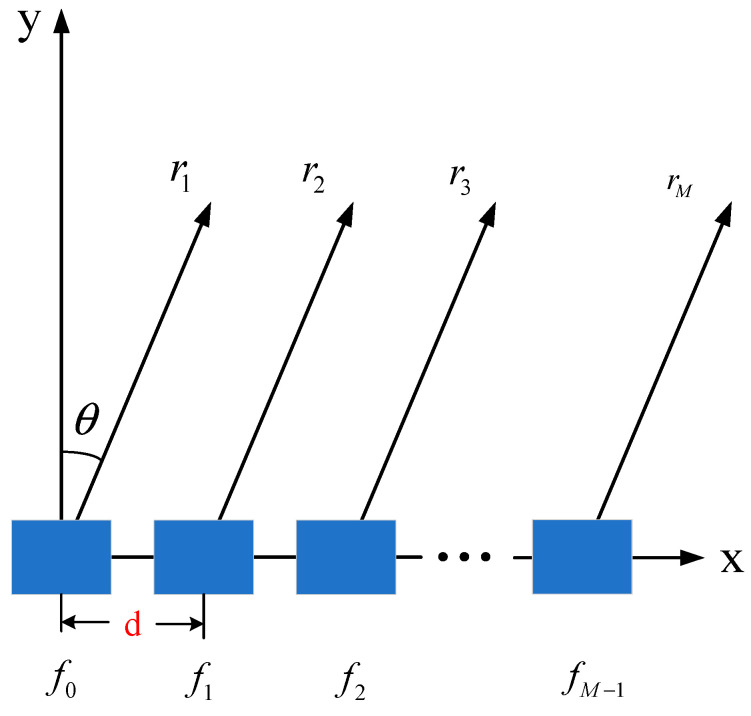
Schematic of the FDA-transmitted array.

As shown in Figure 2, FDA introduces a frequency offset that is significantly smaller than the center frequency to each coherent signal. The radiation frequency of the first array is $f_{0}+\Delta f_{1}$, the radiation frequency of the second array is $f_{0}+\Delta f_{2}$, and the radiation signal frequency of the *m*-th array is:

(4) $$f_{m}=f_{0}+\Delta f_{m}\;\left(\Delta f_{1}=0\right),$$

where $f_{0}$ is the center frequency of each coherent signal. In Figure 2, $r_{1}$ is the distance from the beam pointing position to the first array element (reference array element), and $r_{2}$ is the distance from the beam pointing position to the second array element. Similarly, $r_{M}$ is the distance from the beam pointing position to the *M*-th array element.

If the frequency offset of each array is identical, the frequency of the radiation signal of the *M*-th array is:

(5) $$f_{m}=f_{0}+\left(m-1\right)\cdot \Delta f\;,\;m=1,2,\cdot \cdot \cdot ,M,$$

where *M* represents the array sequence.

Taking a one-dimensional linear uniform array frequency array as an example, the uniformly weighted transmitting pattern is derived as follows.

The transmitted signal is assumed to be:

(6) $$S_{m}\left(t\right)=\exp\left(-j2\pi f_{m}t\right),$$

where $f_{m}$ is the signal frequency of the *m*-th array element.

Therefore, the FDA-MIMO beam direction diagram at $\left(r_{m},\theta \right)$:

(7) $$A\left(t;r\right)=\sum_{m=1}^{M}S_{m}\left(t\right)=\sum_{m=1}^{M}\exp\left[-j2\pi f_{m}\left(t-\frac{r_{m}}{c}\right)\right],$$

where $r_{m}$ is the distance from the beam pointing position to the *m*-th array; *c* represents the speed of light. Since the arrays are evenly spaced, the following equation can be obtained:

(8) $$r_{m}=r_{0}-\left(m-1\right)d\sin\theta .$$

where $r_{0}$ is the distance from the beam pointing position to the first array element (reference array element); *m* is the array number; *d* is the array distance; θ is the pointing angle of the expected beam. After combining Equations (4) and (8), Equation (7) can be further rewritten as:

(9) $$\begin{array}{ll} A\left(t;r,\theta \right) & =\sum_{m=1}^{M}\exp\left[-j2\pi \left(f_{0}+\Delta f_{m}\right)\left(t-\frac{r_{0}-\left(m-1\right)d\sin\theta }{c}\right)\right]\\ & =\sum_{m=1}^{M}\exp\left[-j2\pi \left(f_{0}t-\frac{r_{0}}{c}f_{0}+\frac{\left(m-1\right)d\sin\theta }{c}f_{0}+\Delta f_{m}t-\frac{r_{0}}{c}\Delta f_{m}+\frac{\left(m-1\right)d\sin\theta }{c}\Delta f_{m}\right)\right] \end{array}.$$

where $\Delta f_{m}$ is the frequency offset of the *m*-th array relative to the reference array. In FDA-MIMO technology, the frequency offset of each array is much smaller than the base carrier frequency (the signal frequency of the reference array), namely $\Delta f_{m}\ll f_{0}$. As a result,

(10) $$\frac{\left(m-1\right)d\sin\theta }{c}\Delta f_{m}\ll \frac{\left(m-1\right)d\sin\theta }{c}f_{0}\;\left(m>1\right).$$

Therefore, Equation (9) can be simplified as:

(11) $$\begin{array}{ll} A\left(t;r,\theta \right) & =\sum_{m=1}^{M}\exp\left[-j2\pi \left(f_{0}+\Delta f_{m}\right)\left(t-\frac{r_{0}-\left(m-1\right)d\sin\theta }{c}\right)\right]\\ & =\sum_{m=1}^{M}\exp\left[-j2\pi \left(f_{0}t-\frac{r_{0}}{c}f_{0}+\frac{\left(m-1\right)d\sin\theta }{c}f_{0}+\Delta f_{m}t-\frac{r_{0}}{c}\Delta f_{m}\right)\right] \end{array}.$$

If the frequency deviation increment of each array of FDA-MIMO is distributed evenly, namely

(12) $$\begin{array}{l} \Delta f_{m}=\left(m-1\right)\Delta f\\ f_{m}=f_{0}+\left(m-1\right)\Delta f \end{array}.$$

Then Equation (11) can be rewritten as:

(13) $$\begin{array}{ll} A\left(t;r,\theta \right) & =\sum_{m=1}^{M}\exp\left[-j2\pi \left(f_{0}+\left(m-1\right)\Delta f\right)\left(t-\frac{r_{0}-\left(m-1\right)d\sin\theta }{c}\right)\right]\\ & =\sum_{m=1}^{M}\exp\left[-j2\pi \left(f_{0}t-\frac{r_{0}}{c}f_{0}+\frac{\left(m-1\right)d\sin\theta }{c}f_{0}+\left(m-1\right)\Delta ft-\frac{r_{0}}{c}\left(m-1\right)\Delta f\right)\right]\\ & =\exp\left[-j2\pi \left(f_{0}t-\frac{r_{0}}{c}f_{0}\right)\right]\cdot \sum_{m=1}^{M}\exp\left[-j2\pi \left(m-1\right)\left(\Delta ft-\frac{r_{0}}{c}\Delta f+\frac{d\sin\theta }{c}f_{0}\right)\right] \end{array}.$$

If the module length of the beam function is only considered rather than the phase, then Equation (13) can be simplified as follows:

(14) $$\left|A\left(t;r,\theta \right)\right|=\frac{\sin\left[M\pi \left(\Delta ft-\frac{r_{0}}{c}\Delta f+\frac{d\sin\theta }{c}f_{0}\right)\right]}{\sin\left[\pi \left(\Delta ft-\frac{r_{0}}{c}\Delta f+\frac{d\sin\theta }{c}f_{0}\right)\right]}.$$

See Appendix A for the specific derivation process from Equation (13) to Equation (14).

If the frequency deviation increment of each array element of FDA-MIMO changes irregularly, the beam function can be expressed as:

(15) $$\begin{array}{ll} A\left(t;r,\theta \right) & =\sum_{m=1}^{M}\exp\left[-j2\pi \left(f_{0}+\Delta f_{m}\right)\left(t-\frac{r_{0}-\left(m-1\right)d\sin\theta }{c}\right)\right]\\ & =\sum_{m=1}^{M}\exp\left[-j2\pi \left(f_{0}t-\frac{r_{0}}{c}f_{0}+\frac{\left(m-1\right)d\sin\theta }{c}f_{0}+\Delta f_{m}t-\frac{r_{0}}{c}\Delta f_{m}\right)\right] \end{array}.$$

**Figure 3:**
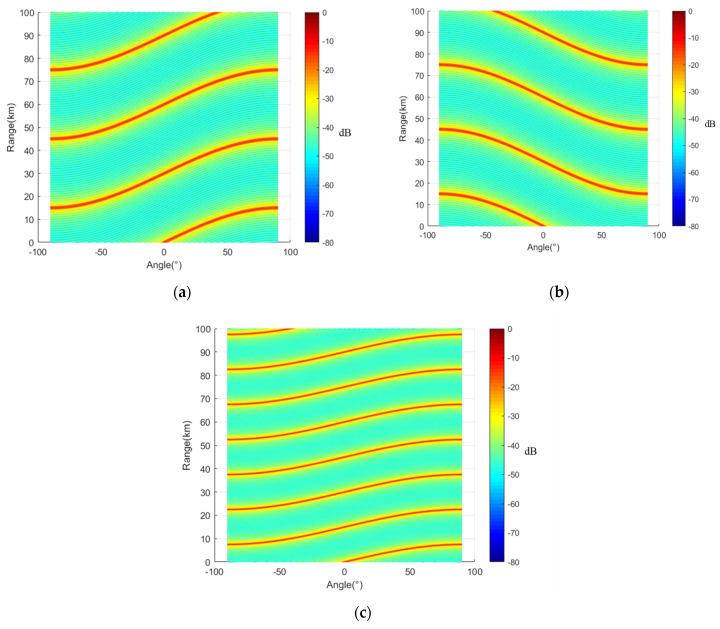
Comparison of FDA-MIMO transmitting beam affected by Δf. (**a**) FDA-MIMO-transmitted beam pattern (frequency offset Δf=10 KHz), the red part represents the crest, and the blue part represents the trough; (**b**) FDA-MIMO-transmitted beam diagram (frequency offset Δf=−10 KHz), the red part represents the crest, and the blue part represents the trough; (**c**) FDA-MIMO-transmitted beam diagram (frequency offset Δf=20 KHz), the red part represents the crest, and the blue part represents the trough.

**Figure 4:**
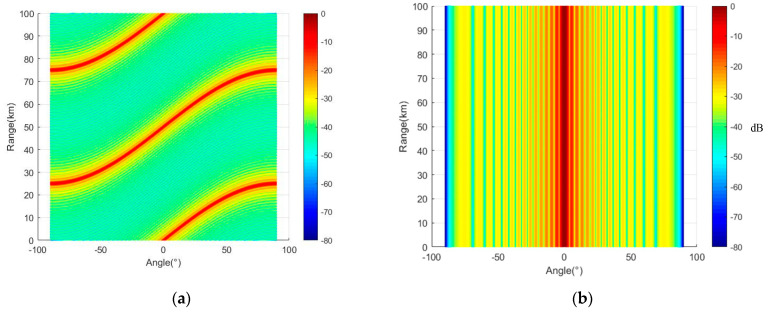
Comparison of array pattern between frequency diverse array and phased array. (**a**) Frequency diverse array; (**b**) phased array.

By analyzing Equations (13) and (15), it can be seen that FDA technology has the following characteristics:

1. As shown in Figure 3, the orientation of the frequency array pattern will be affected by the frequency offset loaded.
2. The most important characteristic of the frequency array is that its array factor is range-dependent. Assuming $\Delta f=3\;\mathrm{kHz},\;M=12,\;f=10\;\mathrm{GHz},\;d\;=\;c/2f_{0}$, Figure 4 compares array orientation diagrams for a frequency array and a phased array. The array pattern of a phased array has no distance dependence, while the unique S-shaped array pattern of the frequency array is caused by its array factor $\Delta f\left(ct-r+d\sin\theta \right)/c$. The distance difference of this factor between θ=0 and θ=π/2 is dΔf/c, where $\lambda_{0}=c/f_{0}$ is the signal wavelength. This indicates that the peaks of its array pattern are a function of $d/f_{0}$ and Δf.

The colors in Figure 2 and Figure 3 represent the amplitude of the beam, with a color bar legend provided on the right side of each figure. Colors that appear closer to red indicate higher beam amplitudes. This visualization was performed using the MATLAB 2018b simulation software. 

## 4. Design Principle of Low Intercept Point-Like Beam

According to the analysis in Section 3, the beam of a radio signal is mainly characterized by a beam function. Therefore, the research on the design principle of a low intercept point-like beam should commence with an in-depth analysis of the beam function. Here, the beam function can be reformulated as follows:

(16) $$\begin{array}{ll} A\left(t;r,\theta \right) & =\sum_{m=1}^{M}\exp\left[-j2\pi \left(f_{0}+\Delta f_{m}\right)\left(t-\frac{r_{0}-\left(m-1\right)d\sin\theta }{c}\right)\right]\\ & =\sum_{m=1}^{M}\exp\left[-j2\pi \left(f_{0}t-\frac{r_{0}}{c}f_{0}+\frac{\left(m-1\right)d\sin\theta }{c}f_{0}+\Delta f_{m}t-\frac{r_{0}}{c}\Delta f_{m}\right)\right] \end{array}.$$

According to Equation (16), once the basic carrier frequency $f_{0}$ and beam synthesis position $\left(r_{m},\theta \right)$ are determined, the amplitude of the beam at different positions is influenced by the array spacing *d* and array frequency offset $\Delta f_{m}$. Considering that, for any given FDA-MIMO system, the array spacing is not modifiable once designed, and it cannot be repeatedly designed and changed many times. Therefore, research on the design principles of low interception point-like beams mainly focuses on the influence of the frequency offset setting of each array element on beam synthesis.

**Figure 5:**
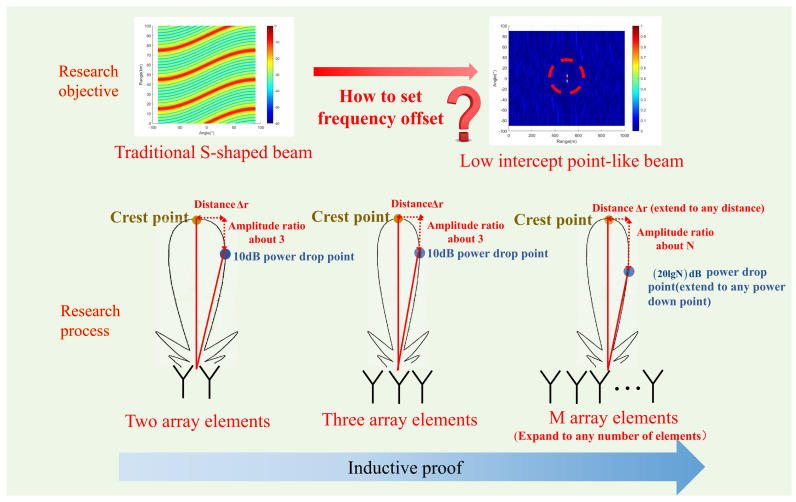
Derivation proof logic diagram.

In this paper, we distinguish between ideal situations and situations with noise and interference and employ the principle of mathematical induction to systematically analyze the influence of frequency offset settings on the synthesis of point beams under the special case of **two array elements**. Then, we extend our analysis to a configuration of **three array elements**. Finally, we deduce the impact of frequency offset settings of each array element on the synthesis of point beams in the case of **M array elements**. The logical diagram of the derivation and proof is shown in Figure 5. Considering that the time variable does not influence the derivation of the entire formula within the beam function, the following formula derivation is carried out at the moment of t = 0. This assumption will not be repeated in the subsequent text.

At the same time, it is necessary to explain the scope of application of the derivation in this paper as follows:

(1) The derivations in this paper are mainly used for close range radio detection probes such as radio fuzes.
(2) Operating frequency: 1 GHz to 40 GHz.
(3) Operation area: radio signals are transmitted from the air to land.
(4) Propagation problem: Because the detection distance of radio fuzes is relatively close, and the use of radio fuzes is usually in an open field, the multipath effect is not obvious. Rain, water vapor, oxygen, etc., can attenuate radio signals. Therefore, the author improves the derivation of this paper from the perspective of signal-to-noise ratio, as detailed in Section 4.2.

### 4.1. Low Intercept Point-Like Beam Design in Ideal Case

#### 4.1.1. Case of Two Array Elements

Assuming that the wave crest point of FDA-MIMO is *H*, then the beam amplitude of *H* is:

(17) $$A_{H_{2}}\left(t;r,\theta \right)=\exp\left[-j2\pi \left(-\frac{r_{0}}{c}f_{0}\right)\right]\cdot \sum_{m=1}^{2}\exp\left[-j2\pi \left(\frac{\left(m-1\right)f_{0}d\sin\theta }{c}-\frac{r_{0}}{c}\Delta f_{m}\right)\right].$$

Then, the beam amplitude at point $H^{\prime }$, Δr away from point *H*, is:

(18) $$A_{H_{2}^{\prime }}\left(t;r,\theta \right)=\exp\left[-j2\pi \left(-\frac{r_{0}+\Delta r}{c}f_{0}\right)\right]\cdot \sum_{m=1}^{2}\exp\left[-j2\pi \left(\frac{\left(m-1\right)f_{0}d\sin\theta }{c}-\frac{r_{0}+\Delta r}{c}\Delta f_{m}\right)\right].$$

Given that $H^{\prime }$ is the 10 dB power drop point, and the distance Δr between *H* and $H^{\prime }$ is very close, the beam will approach the point beam. Therefore,

(19) $$20\mathrm{lg}\left(\frac{A_{H_{2}}}{A_{H_{2}^{\prime }}}\right)=10.$$

Equation (19) is simplified as,

(20) $$\frac{A_{H_{2}}}{A_{H_{2}^{\prime }}}=3.16\approx 3.$$

Therefore,

(21) $$\begin{array}{ll} \frac{A_{H_{2}}}{A_{H_{2}^{\prime }}} & =\frac{\exp\left[-j2\pi \left(-\frac{r_{0}}{c}f_{0}\right)\right]\cdot \sum_{m=1}^{2}\exp\left[-j2\pi \left(\frac{\left(m-1\right)f_{0}d\sin\theta }{c}-\frac{r_{0}}{c}\Delta f_{m}\right)\right]}{\exp\left[-j2\pi \left(-\frac{r_{0}+\Delta r}{c}f_{0}\right)\right]\cdot \sum_{m=1}^{2}\exp\left[-j2\pi \left(\frac{\left(m-1\right)f_{0}d\sin\theta }{c}-\frac{r_{0}+\Delta r}{c}\Delta f_{m}\right)\right]}\\ & =\frac{1+\exp\left[-j2\pi \left(\frac{df_{0}\sin\theta }{c}-\frac{r_{0}}{c}\Delta f_{2}\right)\right]}{\exp\left[-j2\pi \left(-\frac{\Delta r}{c}f_{0}\right)\right]+\exp\left[-j2\pi \left(\frac{df_{0}\sin\theta }{c}-\frac{r_{0}+\Delta r}{c}\Delta f_{2}-\frac{\Delta r}{c}f_{0}\right)\right]}\\ & =3 \end{array}.$$

In fact, the radio detector activates at the terminal phase of the projectile flight, and the angle between the projectile and the ground is usually large and approximately vertical. Therefore, the angle θ between the radio detection beam and the ground normal is very small, sinθ→0. Considering that the radio detector has a range of about 200 m and Δr is very small, $\frac{\Delta r}{c}\to 0$, $\frac{r_{0}}{c}\to 0$, $\frac{r_{0}+\Delta r}{c}\to 0$.

Therefore,

(22) $$-j2\pi \left(\frac{df_{0}\sin\theta }{c}-\frac{r_{0}}{c}\Delta f_{2}\right)\to 0.$$

(23) $$-j2\pi \left(-\frac{\Delta r}{c}f_{0}\right)\to 0.$$

(24) $$-j2\pi \left(\frac{df_{0}\sin\theta }{c}-\frac{r_{0}+\Delta r}{c}\Delta f_{2}-\frac{\Delta r}{c}f_{0}\right)\to 0.$$

Therefore, exp(x) in Equation (21) can be applied to the first-order Talor expansion of the exponential function at *x* = 0, i.e.,

(25) $$\exp\left(x\right)=1+x+ο\left(x\right).$$

Therefore, the first-order Talor expansion of Equation (21) at *x* = 0 is:

(26) $$\begin{array}{ll} \frac{A_{H_{2}}}{A_{H_{2}^{\prime }}} & =\frac{1+1-j2\pi \left(\frac{df_{0}\sin\theta }{c}-\frac{r_{0}}{c}\Delta f_{2}\right)}{1-j2\pi \left(-\frac{\Delta r}{c}f_{0}\right)+1-j2\pi \left(\frac{df_{0}\sin\theta }{c}-\frac{r_{0}+\Delta r}{c}\Delta f_{2}-\frac{\Delta r}{c}f_{0}\right)}\\ & =\frac{2-j2\pi \left(\frac{df_{0}\sin\theta }{c}-\frac{r_{0}}{c}\Delta f_{2}\right)}{2-j2\pi \left(\frac{df_{0}\sin\theta }{c}-\frac{r_{0}+\Delta r}{c}\Delta f_{2}-\frac{2\Delta r}{c}f_{0}\right)} \end{array}.$$

In Equation (21) $\frac{A_{H_{2}}}{A_{H_{2}^{\prime }}}=\frac{\left\|A_{H_{2}}\right\|}{\left\|A_{H_{2}^{\prime }}\right\|}=3$. Therefore, the following equation can be obtained:

(27) $$\begin{array}{ll} \frac{A_{H_{2}}}{A_{H_{2}^{\prime }}} & =\frac{\left\|A_{H_{2}}\right\|}{\left\|A_{H_{2}^{\prime }}\right\|}=\frac{\left\|2-j2\pi \left(\frac{df_{0}\sin\theta }{c}-\frac{r_{0}}{c}\Delta f_{2}\right)\right\|}{\left\|2-j2\pi \left(\frac{df_{0}\sin\theta }{c}-\frac{r_{0}+\Delta r}{c}\Delta f_{2}-\frac{2\Delta r}{c}f_{0}\right)\right\|}\\ & ={\left(\frac{4+\frac{4\pi^{2}}{c^{2}}\left[d^{2}f_{0}^{2}\sin^{2}\theta -2df_{0}\sin\theta r_{0}\Delta f_{2}+r_{0}^{2}\Delta f_{2}^{2}\right]}{4+\frac{4\pi^{2}}{c^{2}}{\left[df_{0}\sin\theta -\left(r_{0}+\Delta r\right)\Delta f-2\Delta rf_{0}\right]}^{2}}\right)}^{\frac{1}{2}}\\ & =3 \end{array}.$$

As a result,

(28) $$\frac{4+\frac{4\pi^{2}}{c^{2}}\left[d^{2}f_{0}^{2}\sin^{2}\theta -2df_{0}\sin\theta r_{0}\Delta f_{2}+r_{0}^{2}\Delta f_{2}^{2}\right]}{4+\frac{4\pi^{2}}{c^{2}}{\left[df_{0}\sin\theta -\left(r_{0}+\Delta r\right)\Delta f-2\Delta rf_{0}\right]}^{2}}=9.$$

(29) $$4+\frac{4\pi^{2}}{c^{2}}\left[d^{2}f_{0}^{2}\sin^{2}\theta -2df_{0}\sin\theta r_{0}\Delta f_{2}+r_{0}^{2}\Delta f_{2}^{2}\right]=36+\frac{36\pi^{2}}{c^{2}}{\left[df_{0}\sin\theta -\left(r_{0}+\Delta r\right)\Delta f-2\Delta rf_{0}\right]}^{2}.$$

The value of $\frac{r_{0}^{2}\Delta f_{2}^{2}}{c^{2}}$ is sufficiently small that it can be considered negligible. Consequently, Equation (29) can be simplified to:

(30) $$4+\frac{4\pi^{2}}{c^{2}}\left[d^{2}f_{0}^{2}\sin^{2}\theta -2df_{0}\sin\theta r_{0}\Delta f_{2}\right]=36+\frac{36\pi^{2}}{c^{2}}\left[d^{2}f_{0}^{2}\sin^{2}\theta -2df_{0}\sin\theta r_{0}\Delta f_{2}-4\Delta rf_{0}^{2}d\sin\theta +4\Delta rf_{0}r_{0}\Delta f_{2}+4\Delta r^{2}f_{0}^{2}\right]$$

The frequency offset $\Delta f_{2}$ can be obtained by simplifying Equation (30):

(31) $$\begin{array}{l} \Delta f_{2}=\frac{9\Delta rf_{0}^{2}d\sin\theta -2d^{2}f_{0}^{2}\sin^{2}\theta -9\Delta r^{2}f_{0}^{2}-\frac{2c^{2}}{\pi^{2}}}{f_{0}r_{0}\left(9\Delta r-4d\sin\theta \right)}\\ \Delta f_{1}=0\;(\mathrm{The}\;\mathrm{first}\;\mathrm{array}\;\mathrm{element}\;\mathrm{is}\;\mathrm{the}\;\mathrm{reference}\;\mathrm{array}\;\mathrm{element}\;\mathrm{with}\;\mathrm{no}\;\mathrm{frequency}\;\mathrm{offset}) \end{array}.$$

In the case of two array elements, when the frequency offset design is calculated according to Equation (31), FDA-MIMO can form a low interception point-like beam.

#### 4.1.2. Case of Three Array Elements

Assuming that the wave crest point of FDA-MIMO is H, then the beam amplitude of H is:

(32) $$A_{H_{3}}\left(t;r,\theta \right)=\exp\left[-j2\pi \left(-\frac{r_{0}}{c}f_{0}\right)\right]\cdot \sum_{m=1}^{3}\exp\left[-j2\pi \left(\frac{\left(m-1\right)f_{0}d\sin\theta }{c}-\frac{r_{0}}{c}\Delta f_{m}\right)\right].$$

Then, the beam amplitude at point $H^{\prime }$, Δr away from point *H*, is:

(33) $$A_{H_{3}^{\prime }}\left(t;r,\theta \right)=\exp\left[-j2\pi \left(-\frac{r_{0}+\Delta r}{c}f_{0}\right)\right]\cdot \sum_{m=1}^{3}\exp\left[-j2\pi \left(\frac{\left(m-1\right)f_{0}d\sin\theta }{c}-\frac{r_{0}+\Delta r}{c}\Delta f_{m}\right)\right].$$

We can reasonably assume that $H^{\prime }$ is the 10 dB power drop point. Given that the distance Δr between point *H* and point $H^{\prime }$ is very small, then the beam will approach the point beam. Therefore,

(34) $$20\mathrm{lg}\left(\frac{A_{H_{3}}}{A_{H_{3}^{\prime }}}\right)=10.$$

Equation (34) is simplified as:

(35) $$\frac{A_{H_{3}}}{A_{H_{3}^{\prime }}}=3.16\approx 3.$$

Therefore,

(36) $$\begin{array}{ll} \frac{A_{H_{3}}}{A_{H_{3}^{\prime }}} & =\frac{\exp\left[-j2\pi \left(-\frac{r_{0}}{c}f_{0}\right)\right]\cdot \sum_{m=1}^{3}\exp\left[-j2\pi \left(\frac{\left(m-1\right)f_{0}d\sin\theta }{c}-\frac{r_{0}}{c}\Delta f_{m}\right)\right]}{\exp\left[-j2\pi \left(-\frac{r_{0}+\Delta r}{c}f_{0}\right)\right]\cdot \sum_{m=1}^{3}\exp\left[-j2\pi \left(\frac{\left(m-1\right)f_{0}d\sin\theta }{c}-\frac{r_{0}+\Delta r}{c}\Delta f_{m}\right)\right]}\\ & =\frac{1+\exp\left[-j2\pi \left(\frac{df_{0}\sin\theta }{c}-\frac{r_{0}}{c}\Delta f_{2}\right)\right]+\exp\left[-j2\pi \left(\frac{2df_{0}\sin\theta }{c}-\frac{r_{0}}{c}\Delta f_{3}\right)\right]}{\exp\left[-j2\pi \left(-\frac{\Delta r}{c}f_{0}\right)\right]\cdot \left\{1+\exp\left[-j2\pi \left(\frac{df_{0}\sin\theta }{c}-\frac{r_{0}+\Delta r}{c}\Delta f_{2}\right)\right]+\exp\left[-j2\pi \left(\frac{2df_{0}\sin\theta }{c}-\frac{r_{0}+\Delta r}{c}\Delta f_{3}\right)\right]\right\}}\\ & =3 \end{array}.$$

With reference to the approximation conditions of the two-element case, exp(*x*) in Equation (36) can use the first-order Talor expansion of the exponential function at *x* = 0. As a result, the first-order Talor expansion of Equation (36) at *x* = 0 can be expressed as follows:

(37) $$\begin{array}{ll} \frac{A_{H_{3}}}{A_{H_{3}^{\prime }}} & =\frac{1+1-j2\pi \left(\frac{df_{0}\sin\theta }{c}-\frac{r_{0}}{c}\Delta f_{2}\right)+1-j2\pi \left(\frac{2df_{0}\sin\theta }{c}-\frac{r_{0}}{c}\Delta f_{3}\right)}{1+j2\pi \left(\frac{\Delta r}{c}f_{0}\right)+1-j2\pi \left(\frac{df_{0}\sin\theta }{c}-\frac{r_{0}+\Delta r}{c}\Delta f_{2}-\frac{\Delta r}{c}f_{0}\right)+1-j2\pi \left(\frac{2df_{0}\sin\theta }{c}-\frac{r_{0}+\Delta r}{c}\Delta f_{3}-\frac{\Delta r}{c}f_{0}\right)}\\ & =\frac{3-j2\pi \left(\frac{3df_{0}\sin\theta }{c}-\frac{r_{0}}{c}\Delta f_{2}-\frac{r_{0}}{c}\Delta f_{3}\right)}{3-j2\pi \left(\frac{3df_{0}\sin\theta }{c}-\frac{r_{0}+\Delta r}{c}\Delta f_{2}-\frac{r_{0}+\Delta r}{c}\Delta f_{3}-\frac{3\Delta r}{c}f_{0}\right)} \end{array}.$$

In Equation (37) $\frac{A_{H_{3}}}{A_{H_{3}^{\prime }}}=\frac{\left\|A_{H_{3}}\right\|}{\left\|A_{H_{3}^{\prime }}\right\|}=3$; therefore,

(38) $$\begin{array}{ll} \frac{A_{H_{3}}}{A_{H_{3}^{\prime }}} & =\frac{\left\|A_{H_{3}}\right\|}{\left\|A_{H_{3}^{\prime }}\right\|}=\frac{\left\|3-j2\pi \left(\frac{3df_{0}\sin\theta }{c}-\frac{r_{0}}{c}\Delta f_{2}-\frac{r_{0}}{c}\Delta f_{3}\right)\right\|}{\left\|3-j2\pi \left(\frac{3df_{0}\sin\theta }{c}-\frac{r_{0}+\Delta r}{c}\Delta f_{2}-\frac{r_{0}+\Delta r}{c}\Delta f_{3}-\frac{3\Delta r}{c}f_{0}\right)\right\|}\\ & ={\left(\frac{9+\frac{4\pi^{2}}{c^{2}}{\left[3df_{0}\sin\theta -r_{0}\Delta f_{2}-r_{0}\Delta f_{3}\right]}^{2}}{9+\frac{4\pi^{2}}{c^{2}}{\left[3df_{0}\sin\theta -r_{0}\Delta f_{2}-r_{0}\Delta f_{3}-3\Delta rf_{0}\right]}^{2}}\right)}^{\frac{1}{2}}\\ & =3 \end{array}.$$

After substituting $\Delta f_{2}$ from Equation (31) into Equation (38) yields, the following equation can be obtained:

(39) $$\begin{array}{l} \Delta f_{3}=\frac{54\Delta rf_{0}^{2}d\sin\theta -24d^{2}f_{0}^{2}\sin^{2}\theta -27\Delta r^{2}f_{0}^{2}-\frac{6c^{2}}{\pi^{2}}-r_{0}\Delta f_{2}f_{0}\left(18\Delta r-16d\sin\theta \right)}{f_{0}r_{0}\left(18\Delta r-16d\sin\theta \right)}\\ \Delta f_{2}=\frac{9\Delta rf_{0}^{2}d\sin\theta -2d^{2}f_{0}^{2}\sin^{2}\theta -9\Delta r^{2}f_{0}^{2}-\frac{2c^{2}}{\pi^{2}}}{f_{0}r_{0}\left(9\Delta r-4d\sin\theta \right)}\\ \Delta f_{1}=0\;(\mathrm{The}\;\mathrm{first}\;\mathrm{array}\;\mathrm{element}\;\mathrm{is}\;\mathrm{the}\;\mathrm{reference}\;\mathrm{array}\;\mathrm{element}\;\mathrm{with}\;\mathrm{no}\;\mathrm{frequency}\;\mathrm{offset}) \end{array}.$$

It can be seen from Equation (39) that in, the case of three-element arrays, when the frequency offset design is calculated according to Equation (39), FDA-MIMO can form a low interception point-like beam.

#### 4.1.3. Case of M Array Elements

Assuming that the wave crest point of FDA-MIMO is point *H*, then the beam amplitude of point *H* is:

(40) $$A_{H_{M}}\left(t;r,\theta \right)=\exp\left[-j2\pi \left(-\frac{r_{0}}{c}f_{0}\right)\right]\cdot \sum_{m=1}^{M}\exp\left[-j2\pi \left(\frac{\left(m-1\right)f_{0}d\sin\theta }{c}-\frac{r_{0}}{c}\Delta f_{m}\right)\right].$$

Then, the beam amplitude at point $H^{\prime }$, Δr away from point *H*, is:

(41) $$A_{H_{M}^{\prime }}\left(t;r,\theta \right)=\exp\left[-j2\pi \left(-\frac{r_{0}+\Delta r}{c}f_{0}\right)\right]\cdot \sum_{m=1}^{M}\exp\left[-j2\pi \left(\frac{\left(m-1\right)f_{0}d\sin\theta }{c}-\frac{r_{0}+\Delta r}{c}\Delta f_{m}\right)\right].$$

In Section 4.1 and Section 4.2, it is assumed that point $H^{\prime }$ is the 10 dB power decline point, which is extended to 20lgN dB, the power decline point in this section. Therefore,

(42) $$\frac{A_{H_{M}}}{A_{H_{M}^{\prime }}}=N,$$

(43) $$\begin{array}{ll} \frac{A_{H_{M}}}{A_{H_{M}^{\prime }}} & =\frac{\exp\left[-j2\pi \left(-\frac{r_{0}}{c}f_{0}\right)\right]\cdot \sum_{m=1}^{M}\exp\left[-j2\pi \left(\frac{\left(m-1\right)f_{0}d\sin\theta }{c}-\frac{r_{0}}{c}\Delta f_{m}\right)\right]}{\exp\left[-j2\pi \left(-\frac{r_{0}+\Delta r}{c}f_{0}\right)\right]\cdot \sum_{m=1}^{M}\exp\left[-j2\pi \left(\frac{\left(m-1\right)f_{0}d\sin\theta }{c}-\frac{r_{0}+\Delta r}{c}\Delta f_{m}\right)\right]}\\ & =\frac{\sum_{m=1}^{M}\exp\left[-j2\pi \left(\frac{\left(m-1\right)f_{0}d\sin\theta }{c}-\frac{r_{0}}{c}\Delta f_{m}\right)\right]}{\exp\left[-j2\pi \left(-\frac{\Delta r}{c}f_{0}\right)\right]\cdot \sum_{m=1}^{M}\exp\left[-j2\pi \left(\frac{\left(m-1\right)f_{0}d\sin\theta }{c}-\frac{r_{0}+\Delta r}{c}\Delta f_{m}\right)\right]}\\ & =N \end{array}.$$

With reference to the approximation conditions of the two-element case, exp(*x*) in Equation (43) can use the first-order Talor expansion of exponential function at *x* = 0, so the first-order Talor expansion of Equation (43) at *x* = 0 is as follows:

(44) $$\frac{A_{H_{M}}}{A_{H_{M}^{\prime }}}=\frac{M-j\frac{2\pi }{c}\left(\frac{M\left(M-1\right)}{2}df_{0}\sin\theta -r_{0}\left(\sum_{m=2}^{M-1}\Delta f_{m}+\Delta f_{M}\right)\right)}{M-j\frac{2\pi }{c}\left[\frac{M\left(M-1\right)}{2}df_{0}\sin\theta -r_{0}\left(\sum_{m=2}^{M-1}\Delta f_{m}+\Delta f_{M}\right)-M\Delta rf_{0}\right]}=N.$$

Given that $\frac{A_{H_{M}}}{A_{H_{M}^{\prime }}}=\frac{\left\|A_{H_{M}}\right\|}{\left\|A_{H_{M}^{\prime }}\right\|}=N$ in Equation (44),

(45) $$\begin{array}{ll} \frac{A_{H_{M}}}{A_{H_{M}^{\prime }}}= & \frac{\left\|A_{H_{M}}\right\|}{\left\|A_{H_{M}^{\prime }}\right\|}=\frac{\left\|M-j\frac{2\pi }{c}\left(\frac{M\left(M-1\right)}{2}df_{0}\sin\theta -r_{0}\left(\sum_{m=2}^{M-1}\Delta f_{m}+\Delta f_{M}\right)\right)\right\|}{\left\|M-j\frac{2\pi }{c}\left[\frac{M\left(M-1\right)}{2}df_{0}\sin\theta -r_{0}\left(\sum_{m=2}^{M-1}\Delta f_{m}+\Delta f_{M}\right)-M\Delta rf_{0}\right]\right\|}\\ & ={\left(\frac{M^{2}+\frac{4\pi^{2}}{c^{2}}{\left[\frac{M\left(M-1\right)}{2}df_{0}\sin\theta -r_{0}\left(\sum_{m=2}^{M-1}\Delta f_{m}+\Delta f_{M}\right)\right]}^{2}}{M^{2}+\frac{4\pi^{2}}{c^{2}}{\left[\frac{M\left(M-1\right)}{2}df_{0}\sin\theta -r_{0}\left(\sum_{m=2}^{M-1}\Delta f_{m}+\Delta f_{M}\right)-M\Delta rf_{0}\right]}^{2}}\right)}^{\frac{1}{2}}\\ & =N \end{array}.$$

By solving Equation (45), we can obtain:

(46) $$\begin{array}{l} \Delta f_{M}=\frac{\frac{4N^{2}\pi^{2}}{c^{2}}\left[M^{2}\left(M-1\right)df_{0}^{2}\sin\theta \Delta r-M^{2}\Delta r^{2}f_{0}^{2}\right]-\left(N^{2}-1\right)M^{2}-\frac{M^{2}{\left(M-1\right)}^{2}\left(N^{2}-1\right)\pi^{2}}{c^{2}}d^{2}f_{0}^{2}\sin^{2}\theta }{r_{0}f_{0}\left(\frac{8N^{2}\pi^{2}}{c^{2}}M\Delta r-\frac{\left(N^{2}-1\right)4\pi^{2}M\left(M-1\right)d\sin\theta }{c^{2}}\right)}\\ -\Delta f_{M-1}-\sum_{m=2}^{M-2}\Delta f_{m} \end{array}.$$

In the same way,

(47) $$\begin{array}{l} \Delta f_{M-1}=\frac{\frac{4N^{2}\pi^{2}}{c^{2}}\left[\left(M-2\right){\left(M-1\right)}^{2}df_{0}^{2}\sin\theta \Delta r-{\left(M-1\right)}^{2}\Delta r^{2}f_{0}^{2}\right]-\left(N^{2}-1\right){\left(M-1\right)}^{2}-\frac{{\left(M-2\right)}^{2}{\left(M-1\right)}^{2}\left(N^{2}-1\right)\pi^{2}}{c^{2}}d^{2}f_{0}^{2}\sin^{2}\theta }{r_{0}f_{0}\left(\frac{8N^{2}\pi^{2}}{c^{2}}\left(M-1\right)\Delta r-\frac{\left(N^{2}-1\right)4\pi^{2}\left(M-2\right)\left(M-1\right)d\sin\theta }{c^{2}}\right)}\\ -\sum_{m=2}^{M-2}\Delta f_{m} \end{array}.$$

After substituting Equation (47) into Equation (46), it can be obtained that:

(48) $$\begin{array}{ll} \Delta f_{m} & =\frac{-\frac{1}{\pi^{2}}\left[4\left(N^{2}-1\right)\left(M-1\right)Mc^{2}\right]\left[\left(N^{2}-1\right)d\sin\theta +2N^{2}\Delta r\right]}{r_{0}f_{0}\left[8N^{2}M\Delta r-\left(N^{2}-1\right)4M\left(M-1\right)d\sin\theta \right]\left[8N^{2}\left(M-1\right)\Delta r-\left(N^{2}-1\right)4\left(M-1\right)\left(M-2\right)d\sin\theta \right]}\\ & +\frac{d^{2}f_{0}^{2}\sin^{2}\theta \left[8N^{2}\left(N^{2}-1\right){\left(M-1\right)}^{2}M\left(-5M+8\right)\Delta r+8{\left(N^{2}-1\right)}^{2}M{\left(M-1\right)}^{3}\left(M-2\right)d\sin\theta \right]}{r_{0}f_{0}\left[8N^{2}M\Delta r-\left(N^{2}-1\right)4M\left(M-1\right)d\sin\theta \right]\left[8N^{2}\left(M-1\right)\Delta r-\left(N^{2}-1\right)4\left(M-1\right)\left(M-2\right)d\sin\theta \right]}\\ & +\frac{\Delta r^{2}f_{0}^{2}\left\{16N^{2}M\left(M-1\right)d\sin\theta \left[4N^{2}M-5N^{2}+1\right]-32N^{4}M\left(M-1\right)\Delta r\right\}}{r_{0}f_{0}\left[8N^{2}M\Delta r-\left(N^{2}-1\right)4M\left(M-1\right)d\sin\theta \right]\left[8N^{2}\left(M-1\right)\Delta r-\left(N^{2}-1\right)4\left(M-1\right)\left(M-2\right)d\sin\theta \right]} \end{array}.$$

Furthermore, if the distance between the peak point *H* and the power decline point $H^{\prime }$ is r (r≫Δr), Equation (48) needs to be integrated with Δr as the principal element. However, when *r* is relatively large, the beam can no longer be approximated as a point source and falls outside the scope of this study. Therefore, this paper will no longer solve the frequency offset expression when the distance between two points is *r*.

### 4.2. Low Interception Point-Like Beam Design with Noise and Interference

In this section, the design principle of low interception point-like beam with noise and interference is mainly derived and the propagation of the beam in the Gaussian channel is analyzed. Considering the propagation of the beam in the Gaussian channel, the Shannon formula can accurately reflect the relationship between channel capacity, channel bandwidth, and signal-to-noise ratio. Shannon’s formula is shown in Equation (49).

(49) $$C=B\log_{2}\left(1+\frac{S}{N}\right),$$

where *C* is the channel capacity. *B* is the channel bandwidth. *S* is the average power of the signal and *N* is the average power of the noise. *S*/*N* is the signal-to-noise ratio. In the derivation of this section, the Shannon formula is used for reference, and Shannon weighting is carried out on the amplitude of the beam; the weight is $\log_{2}\left(1+\frac{S}{N}\right)$.

#### 4.2.1. Case of Two Array Elements

When considering noise and interference, the amplitude of H point is weighted by Shannon:

(50) $$A_{JHS_{2}}\left(t;r,\theta \right)=\exp\left[-j2\pi \left(-\frac{r_{0}}{c}f_{0}\right)\right]\cdot \sum_{m=1}^{2}\exp\left[-j2\pi \left(\frac{\left(m-1\right)f_{0}d\sin\theta }{c}-\frac{r_{0}}{c}\Delta f_{m}\right)\right]\cdot \log_{2}\left(1+\frac{S}{N}\right).$$

Then, the beam amplitude of point $H^{\prime }$ at a distance Δr to point H is:

(51) $$A_{JH^{\prime }S_{2}}\left(t;r,\theta \right)=\exp\left[-j2\pi \left(-\frac{r_{0}+\Delta r}{c}f_{0}\right)\right]\cdot \sum_{m=1}^{2}\exp\left[-j2\pi \left(\frac{\left(m-1\right)f_{0}d\sin\theta }{c}-\frac{r_{0}+\Delta r}{c}\Delta f_{m}\right)\right]\cdot \log_{2}\left(1+\frac{S}{N}\right).$$

Given that $H^{\prime }$ is the 10 dB power drop point, and the distance Δr between *H* and $H^{\prime }$ is very close, the beam will approach the point beam. Therefore,

(52) $$20\mathrm{lg}\left(\frac{A_{JHS_{2}}}{A_{JH^{\prime }S_{2}}}\right)=10.$$

Equation (52) is simplified as:

(53) $$\frac{A_{JHS_{2}}}{A_{JH^{\prime }S_{2}}}=3.16\approx 3.$$

Therefore,

(54) $$\begin{array}{ll} \frac{A_{JHS_{2}}}{A_{JH^{\prime }S_{2}}} & =\frac{\exp\left[-j2\pi \left(-\frac{r_{0}}{c}f_{0}\right)\right]\cdot \sum_{m=1}^{2}\exp\left[-j2\pi \left(\frac{\left(m-1\right)f_{0}d\sin\theta }{c}-\frac{r_{0}}{c}\Delta f_{m}\right)\right]\cdot \log_{2}\left(1+\frac{S}{N}\right)}{\exp\left[-j2\pi \left(-\frac{r_{0}+\Delta r}{c}f_{0}\right)\right]\cdot \sum_{m=1}^{2}\exp\left[-j2\pi \left(\frac{\left(m-1\right)f_{0}d\sin\theta }{c}-\frac{r_{0}+\Delta r}{c}\Delta f_{m}\right)\right]\cdot \log_{2}\left(1+\frac{S}{N}\right)}\\ & =\frac{1+\exp\left[-j2\pi \left(\frac{df_{0}\sin\theta }{c}-\frac{r_{0}}{c}\Delta f_{2}\right)\right]}{\exp\left[-j2\pi \left(-\frac{\Delta r}{c}f_{0}\right)\right]+\exp\left[-j2\pi \left(\frac{df_{0}\sin\theta }{c}-\frac{r_{0}+\Delta r}{c}\Delta f_{2}-\frac{\Delta r}{c}f_{0}\right)\right]}\\ & =3 \end{array}.$$

It can be seen from Equation (54) that the subsequent derivation is the same as that in Section 4.1.1. The author will not repeat; we can calculate the frequency offset $\Delta f_{2}$:

(55) $$\begin{array}{l} \Delta f_{2}=\frac{9\Delta rf_{0}^{2}d\sin\theta -2d^{2}f_{0}^{2}\sin^{2}\theta -9\Delta r^{2}f_{0}^{2}-\frac{2c^{2}}{\pi^{2}}}{f_{0}r_{0}\left(9\Delta r-4d\sin\theta \right)}\\ \Delta f_{1}=0\;(\mathrm{The}\;\mathrm{first}\;\mathrm{array}\;\mathrm{element}\;\mathrm{is}\;\mathrm{the}\;\mathrm{reference}\;\mathrm{array}\;\mathrm{element}\;\mathrm{with}\;\mathrm{no}\;\mathrm{frequency}\;\mathrm{offset}) \end{array}.$$

#### 4.2.2. Case of M Array Elements

In the presence of noise and interference, assuming that the peak point of the FDA-MIMO received by the fuze jammer is still H point, then the H-point beam amplitude weighted by the signal-to-interference noise ratio is:

(56) $$A_{JHS_{M}}\left(t;r,\theta \right)=\exp\left[-j2\pi \left(-\frac{r_{0}}{c}f_{0}\right)\right]\cdot \sum_{m=1}^{M}\exp\left[-j2\pi \left(\frac{\left(m-1\right)f_{0}d\sin\theta }{c}-\frac{r_{0}}{c}\Delta f_{m}\right)\right]\cdot \log_{2}\left(1+\frac{S}{N}\right).$$

After the signal-to-interference noise ratio is weighted, the beam amplitude of the $H^{\prime }$ point from the H point Δr is:

(57) $$A_{JH^{\prime }S_{M}}\left(t;r,\theta \right)=\exp\left[-j2\pi \left(-\frac{r_{0}+\Delta r}{c}f_{0}\right)\right]\cdot \sum_{m=1}^{M}\exp\left[-j2\pi \left(\frac{\left(m-1\right)f_{0}d\sin\theta }{c}-\frac{r_{0}+\Delta r}{c}\Delta f_{m}\right)\right]\cdot \log_{2}\left(1+\frac{S}{N}\right).$$

Given that $H^{\prime }$ is the 20lgN dB power drop point, and the distance Δr between *H* and $H^{\prime }$ is very close, the beam will approach the point beam. Therefore,

(58) $$\frac{A_{JHS_{M}}}{A_{JH^{\prime }S_{M}}}=N.$$

(59) $$\begin{array}{ll} \frac{A_{JHS_{M}}}{A_{JH^{\prime }S_{M}}} & =\frac{\exp\left[-j2\pi \left(-\frac{r_{0}}{c}f_{0}\right)\right]\cdot \sum_{m=1}^{M}\exp\left[-j2\pi \left(\frac{\left(m-1\right)f_{0}d\sin\theta }{c}-\frac{r_{0}}{c}\Delta f_{m}\right)\right]\cdot \log_{2}\left(1+\frac{S}{N}\right)}{\exp\left[-j2\pi \left(-\frac{r_{0}+\Delta r}{c}f_{0}\right)\right]\cdot \sum_{m=1}^{M}\exp\left[-j2\pi \left(\frac{\left(m-1\right)f_{0}d\sin\theta }{c}-\frac{r_{0}+\Delta r}{c}\Delta f_{m}\right)\right]\cdot \log_{2}\left(1+\frac{S}{N}\right)}\\ & =\frac{\sum_{m=1}^{M}\exp\left[-j2\pi \left(\frac{\left(m-1\right)f_{0}d\sin\theta }{c}-\frac{r_{0}}{c}\Delta f_{m}\right)\right]}{\exp\left[-j2\pi \left(-\frac{\Delta r}{c}f_{0}\right)\right]\cdot \sum_{m=1}^{M}\exp\left[-j2\pi \left(\frac{\left(m-1\right)f_{0}d\sin\theta }{c}-\frac{r_{0}+\Delta r}{c}\Delta f_{m}\right)\right]}\\ & =N \end{array}.$$

With reference to the approximation conditions of the two-element case, exp(*x*) in Equation (59) can use the first-order Talor expansion of exponential function at *x* = 0, so the first-order Talor expansion of Equation (59) at *x* = 0 is as follows:

(60) $$\frac{A_{JHS_{M}}}{A_{JH^{\prime }S_{M}}}=\frac{M-j\frac{2\pi }{c}\left(\frac{M\left(M-1\right)}{2}df_{0}\sin\theta -r_{0}\left(\sum_{m=2}^{M-1}\Delta f_{m}+\Delta f_{M}\right)\right)}{M-j\frac{2\pi }{c}\left[\frac{M\left(M-1\right)}{2}df_{0}\sin\theta -r_{0}\left(\sum_{m=2}^{M-1}\Delta f_{m}+\Delta f_{M}\right)-M\Delta rf_{0}\right]}=N.$$

Given that $\frac{A_{JHS_{M}}}{A_{JH^{\prime }S_{M}}}=\frac{\left\|A_{JHS_{M}}\right\|}{\left\|A_{JH^{\prime }S_{M}}\right\|}=N$ in Equation (60),

(61) $$\begin{array}{ll} \frac{A_{JHS_{M}}}{A_{JH^{\prime }S_{M}}} & =\frac{\left\|A_{JHS_{M}}\right\|}{\left\|A_{JH^{\prime }S_{M}}\right\|}=\frac{\left\|M-j\frac{2\pi }{c}\left(\frac{M\left(M-1\right)}{2}df_{0}\sin\theta -r_{0}\left(\sum_{m=2}^{M-1}\Delta f_{m}+\Delta f_{M}\right)\right)\right\|}{\left\|M-j\frac{2\pi }{c}\left[\frac{M\left(M-1\right)}{2}df_{0}\sin\theta -r_{0}\left(\sum_{m=2}^{M-1}\Delta f_{m}+\Delta f_{M}\right)-M\Delta rf_{0}\right]\right\|}\\ & ={\left(\frac{M^{2}+\frac{4\pi^{2}}{c^{2}}{\left[\frac{M\left(M-1\right)}{2}df_{0}\sin\theta -r_{0}\left(\sum_{m=2}^{M-1}\Delta f_{m}+\Delta f_{M}\right)\right]}^{2}}{M^{2}+\frac{4\pi^{2}}{c^{2}}{\left[\frac{M\left(M-1\right)}{2}df_{0}\sin\theta -r_{0}\left(\sum_{m=2}^{M-1}\Delta f_{m}+\Delta f_{M}\right)-M\Delta rf_{0}\right]}^{2}}\right)}^{\frac{1}{2}}\\ & =N \end{array}.$$

By solving Equation (61), we can obtain:

(62) $$\begin{array}{l} \Delta f_{M}=\frac{\frac{4N^{2}\pi^{2}}{c^{2}}\left[M^{2}\left(M-1\right)df_{0}^{2}\sin\theta \Delta r-M^{2}\Delta r^{2}f_{0}^{2}\right]-\left(N^{2}-1\right)M^{2}-\frac{M^{2}{\left(M-1\right)}^{2}\left(N^{2}-1\right)\pi^{2}}{c^{2}}d^{2}f_{0}^{2}\sin^{2}\theta }{r_{0}f_{0}\left(\frac{8N^{2}\pi^{2}}{c^{2}}M\Delta r-\frac{\left(N^{2}-1\right)4\pi^{2}M\left(M-1\right)d\sin\theta }{c^{2}}\right)}\\ -\Delta f_{M-1}-\sum_{m=2}^{M-2}\Delta f_{m} \end{array}.$$

In the same way,

(63) $$\begin{array}{l} \Delta f_{M-1}=\frac{\frac{4N^{2}\pi^{2}}{c^{2}}\left[\left(M-2\right){\left(M-1\right)}^{2}df_{0}^{2}\sin\theta \Delta r-{\left(M-1\right)}^{2}\Delta r^{2}f_{0}^{2}\right]-\left(N^{2}-1\right){\left(M-1\right)}^{2}-\frac{{\left(M-2\right)}^{2}{\left(M-1\right)}^{2}\left(N^{2}-1\right)\pi^{2}}{c^{2}}d^{2}f_{0}^{2}\sin^{2}\theta }{r_{0}f_{0}\left(\frac{8N^{2}\pi^{2}}{c^{2}}\left(M-1\right)\Delta r-\frac{\left(N^{2}-1\right)4\pi^{2}\left(M-2\right)\left(M-1\right)d\sin\theta }{c^{2}}\right)}\\ -\sum_{m=2}^{M-2}\Delta f_{m} \end{array}.$$

After substituting Equation (63) into Equation (62), it can be obtained that:

(64) $$\begin{array}{ll} \Delta f_{m} & =\frac{-\frac{1}{\pi^{2}}\left[4\left(N^{2}-1\right)\left(M-1\right)Mc^{2}\right]\left[\left(N^{2}-1\right)d\sin\theta +2N^{2}\Delta r\right]}{r_{0}f_{0}\left[8N^{2}M\Delta r-\left(N^{2}-1\right)4M\left(M-1\right)d\sin\theta \right]\left[8N^{2}\left(M-1\right)\Delta r-\left(N^{2}-1\right)4\left(M-1\right)\left(M-2\right)d\sin\theta \right]}\\ & +\frac{d^{2}f_{0}^{2}\sin^{2}\theta \left[8N^{2}\left(N^{2}-1\right){\left(M-1\right)}^{2}M\left(-5M+8\right)\Delta r+8{\left(N^{2}-1\right)}^{2}M{\left(M-1\right)}^{3}\left(M-2\right)d\sin\theta \right]}{r_{0}f_{0}\left[8N^{2}M\Delta r-\left(N^{2}-1\right)4M\left(M-1\right)d\sin\theta \right]\left[8N^{2}\left(M-1\right)\Delta r-\left(N^{2}-1\right)4\left(M-1\right)\left(M-2\right)d\sin\theta \right]}\\ & +\frac{\Delta r^{2}f_{0}^{2}\left\{16N^{2}M\left(M-1\right)d\sin\theta \left[4N^{2}M-5N^{2}+1\right]-32N^{4}M\left(M-1\right)\Delta r\right\}}{r_{0}f_{0}\left[8N^{2}M\Delta r-\left(N^{2}-1\right)4M\left(M-1\right)d\sin\theta \right]\left[8N^{2}\left(M-1\right)\Delta r-\left(N^{2}-1\right)4\left(M-1\right)\left(M-2\right)d\sin\theta \right]} \end{array}.$$

By comparison with Equations (48) and (64), it can be seen that, in the process of formula derivation, the frequency offset setting formula for the optimal convergence of the FDA-MIMO spot beam is the same regardless of whether noise and external interference are considered, because, in the process of dividing the amplitude, the weight of the signal-to-noise ratio to the amplitude has been reduced in the numerator and denominator (as shown in Equation (64)). In fact, the beam peak H point and the power drop point $H^{\prime }$ are always in the same environment. The effect of SNR on H point and point $H^{\prime }$ is the same. Therefore, it is true and credible that the weight of the signal-to-noise ratio to the amplitude is canceled when the numerator and denominator are divided.

### 4.3. Physical Explanation of Point-Beam Design Principle and Related Inferences

Through the analysis of the FDA-MIMO beam function, the amplitude of the beam at different positions is influenced by the array spacing d and the array frequency offset $\Delta f_{m}$ when the basic carrier frequency $f_{0}$ and the beam synthesis position $\left(r_{m},\theta \right)$ are determined. Given the constraints imposed by the hardware system, the array spacing *d* cannot be modified or changed repeatedly. Therefore, research on the design principle of low interception point-like beams mainly focuses on the influence of the frequency offset setting of each array on the beam synthesis.

**Figure 6:**
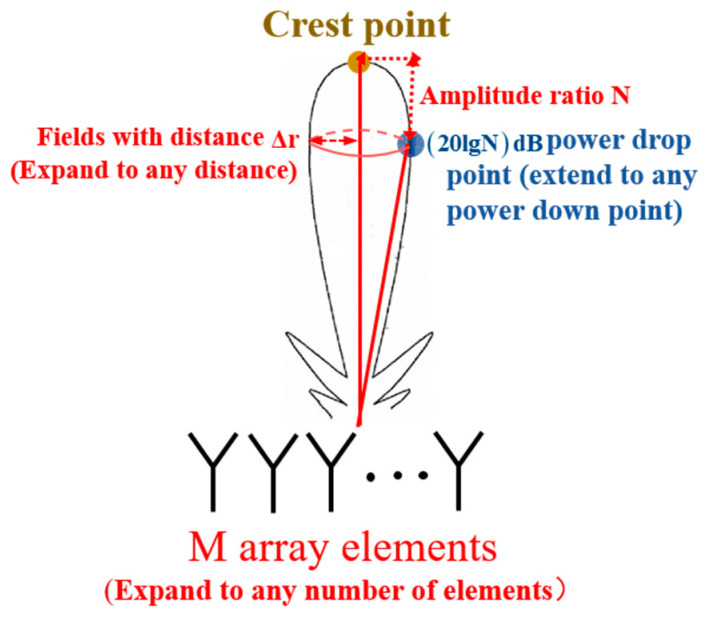
Schematic diagram of low intercept point-like beam design principle.

In the frequency offset setting of each array element, by setting the peak point *H* and the 10 dB power drop point $H^{\prime }$ in a relatively close range (Δr), the goal is to achieve a significant beam amplitude in a small vicinity around point *H* while ensuring a rapid attenuation of the beam amplitude in other regions. Further, the beam function is used to calculate the frequency offset of each array element, thereby establishing the design principle of a low interception point-like beam. The schematic diagram of the low intercept point-like beam design principle is shown in Figure 6.

**Inference** **1.**  *The frequency offset of each FDA-MIMO array is gradually increasing.*

The proof of Inference 1 is shown in Appendix B.

**Inference** **2.**  *Based on Equation (48), the frequency offset of each array element of FDA-MIMO forming a point beam has a frequency deviation fluctuation of 10%.*

The proof of Inference 2 is shown in Appendix C.

## 5. Simulation and Discussion

In order to further demonstrate the feasibility of the point-like beam design principle, this section conducts simulations and discussions from three perspectives. ① The converging performance of the point-like beam is evaluated by analyzing the transmitting beam pattern and peak sidelobe ratio, range-dimension half-power beamwidth, and angle-dimension half-power beamwidth. ② The low intercept performance of the point-like beam is analyzed from an entropy perspective. ③ The performance of low intercept in the airspace of radio detectors is simulated and analyzed to evaluate its practical application in electronic countermeasures. All simulations are conducted on a Windows 10 operating system with an Inter(R) Core(TM) i7-9750H CPU using MATLAB 2018b. 

### 5.1. Multi-Dimensional Analysis of the Beam Pattern Formed by the Method Proposed in This Paper

In the simulation of this section, a uniform linear array structure is employed, with specific simulation parameters detailed in Table 1.

**Figure 7:**
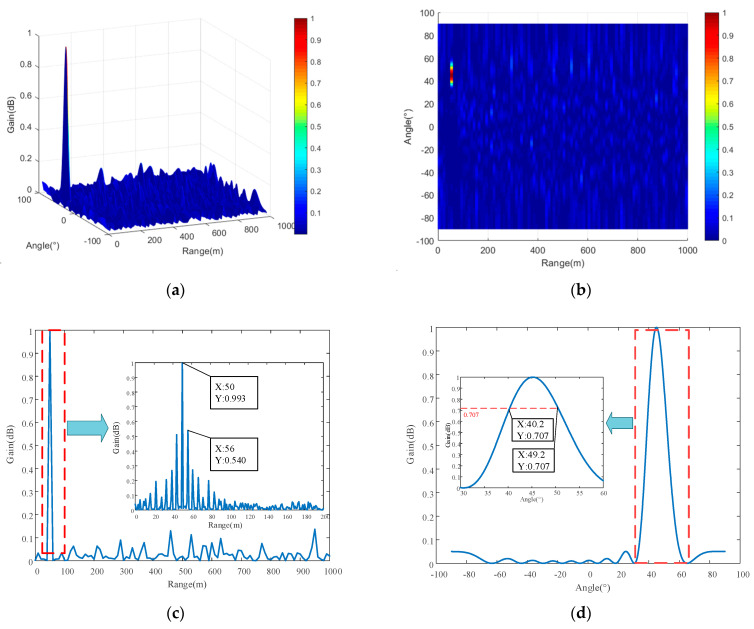
Three-dimensional beam diagrams and their top and section views. (**a**) Three-dimensional view of the beam; (**b**) two-dimensional top view of the beam; (**c**) cutaway view of the beam in the distance dimension; (**d**) cutaway view of the beam in the angular dimension.

It should be noted that, although the frequency offsets of each array mentioned in Table 1 are negative, this does not impact beam synthesis relative to positive frequency offsets. The negative frequency offset of each array element only causes the direction of the beam to be opposite to the bending direction of the beam formed by the positive frequency offset, as illustrated in Figure 3a,b. Therefore, the negative frequency offsets of each array in Table 1 progressively increase in magnitude, and the negative sign only affects the bending direction of the beam. The three-dimensional and two-dimensional top view of the beam and the section diagram of the beam in distance and angle dimensions are shown in Figure 7.

As illustrated in Figure 7a,b, the frequency offset calculated for each array element of the FDA-MIMO system using the point-like beam design principle proposed in this paper effectively eliminates the periodicity of the beam, resulting in a point-like beam with excellent convergence. Figure 7c,d show that, in the distance dimension, the point-like beam is relatively narrow, with a beam width of approximately 1 m. In the angular dimension, the beam width is about 9 degrees.

### 5.2. Comparison and Analysis of Beampattern Formed with Other Methods

**Figure 8:**
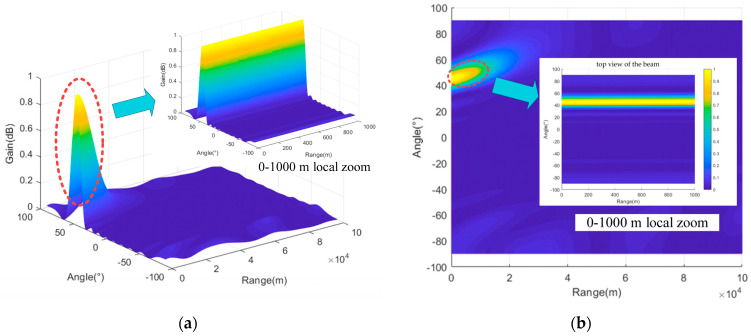
FDA-MIMO beam diagram (logarithmic frequency offset). (**a**) Three-dimensional view of the beam; (**b**) two-dimensional top view of the beam.

**Figure 9:**
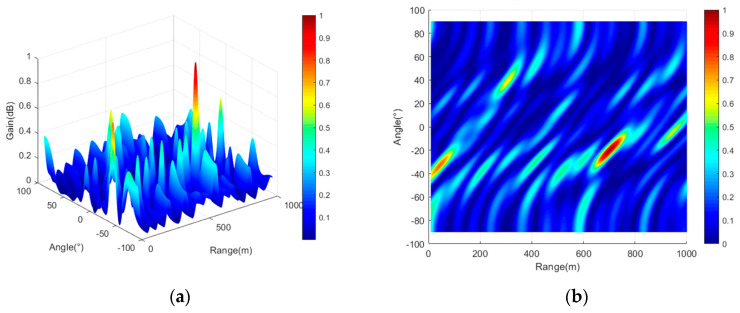
FDA-MIMO beam diagram (cubic frequency offset). (**a**) Three-dimensional view of the beam; (**b**) two-dimensional top view of the beam.

**Figure 10:**
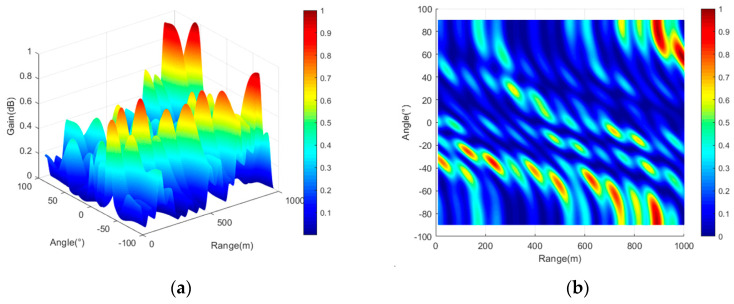
FDA-MIMO beam diagram (reciprocal frequency offset). (**a**) Three-dimensional view of the beam; (**b**) two-dimensional top view of the beam.

**Figure 11:**
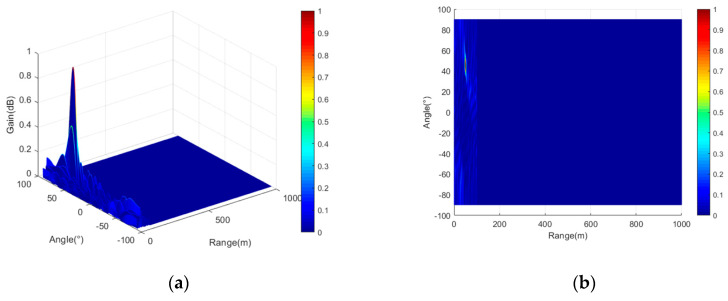
FDA-MIMO beam diagram (proposed in this paper). (**a**) Three-dimensional view of the beam; (**b**) two-dimensional top view of the beam.

To directly demonstrate that the frequency offset setting proposed in this paper enables FDA-MIMO to better form a point-like beam, three typical frequency offset setting methods (logarithmic frequency offset, cubic exponential frequency offset, and reciprocal frequency offset) are compared in this section. In the case of different frequency offset settings, the array structure remains consistent as a uniform linear array. Three typical frequency offset setting formulas are shown in Equations (65)–(67), simulation parameters are shown in Table 2, and three-dimensional beam diagrams are shown in Figure 8, Figure 9, Figure 10 and Figure 11. Considering the detection distance of the radio detector, the simulation distance of the detection beam of the radio detector is usually within 1000 m. In the case of logarithmic frequency deviation, the beam within 1000 m does not converge and remains straight over this distance. To ensure clarity and avoid any potential ambiguity, this section first conducts a simulation over a distance of 10 km. Subsequently, the beam within 1000 m is locally amplified to enhance the persuasiveness of the results.

(65) $$\Delta f_{m}=\log\left(k+1\right)\cdot \Delta f\;\left(k=0,1,2,\cdot \cdot \cdot ,M-1\right)$$

(66) $$\Delta f_{m}={\left(k+1\right)}^{3}\cdot \Delta f\;\left(k=0,1,2,\cdot \cdot \cdot ,M-1\right)$$

(67) $$\Delta f_{m}={\left(k+1\right)}^{-1}\cdot \Delta f\;\left(k=0,1,2,\cdot \cdot \cdot ,M-1\right)$$

It can be seen from Figure 8, Figure 9, Figure 10 and Figure 11 that the performance of FDA-MIMO beam pooling with log-frequency bias is poor. In the distance dimension, the beam width reaches about 16 km. In the case of reciprocal frequency offset, there are several high-energy sidelobes in the beam, leading to increased detection errors and weak anti-interference performance. In the cubic exponential frequency offset case, the performance of beam convergence is better than that of log frequency offset and reciprocal frequency offset. However, it still exhibits high-energy sidelobes, which adversely affect the detection accuracy and anti-jamming capabilities of radio detectors. The method proposed in this paper has the best performance of FDA-MIMO beam convergence without high-energy sidelobes. Consequently, the radio detector can detect the target with high precision and achieve low interception and anti-jamming.

### 5.3. Comparative Analysis of Point-Like Beam Performance

To more accurately evaluate the effectiveness of the point-beam design principle proposed in this paper, three typical frequency offset setting methods, Log-FDA, cubed exponential frequency offset (Cub-FDA), and reciprocal frequency offset (Rec-FDA), are selected and compared with the proposed method. The comparison involves three aspects: peak-to-sidelobe ratio (PSR), range-dimension half power bandwidth (HPBW_r_), and angle-dimension half power beamwidth (HPBW_θ_). The numerical results of the four frequency offset setting methods at the expected position of the beam are shown in Table 3.

It can be seen from Table 3 that the proposed method has the lowest beam-peak sidelobe ratio index. This indicates that the FDA-MIMO system has a lower sidelobe level when it is set according to the frequency offset calculated by the point-like beam design principle in this paper. In terms of the range-dimension half-beam width, the proposed method can achieve a beam width as narrow as 1 m, fully satisfying the stringent requirements of radio detectors for precise ranging. However, the beam formed by other methods is much larger than 1 m in the distance-dimension half-beam width, which does not meet the working requirements of radio detectors. In terms of the angular-dimension half-beam width, the beam formed by the proposed method is 9 degrees, which is roughly in line with the working requirements of radio detectors. Therefore, the FDA-MIMO system is set according to the frequency offset calculated by the point-beam design principle, resulting in optimal beam convergence performance that meets the operational requirements of radio detectors.

### 5.4. Simulation of Low Intercept Performance Based on Beam Entropy

(a) The frequency offset parameters used in the proposed method are calculated based on Equation (48).

**Figure 12:**
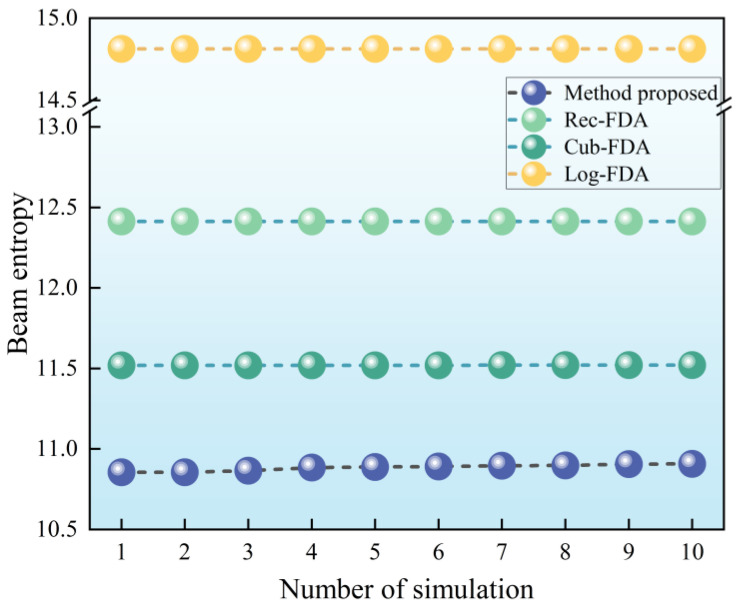
Beam entropy simulation results.

The simulation parameters in this section are the same as those in Section 5.2. In order to ensure that the simulation results have broad applicability, Inference 2 is applied in this section to introduce a 10% random fluctuation of frequency offset. The simulation is performed 500 times, and the minimum entropy value of 10 times is selected from the 500 times to draw the beam entropy simulation result diagram, as shown in Figure 12. Subsequently, the minimum beam entropy of each frequency offset setting is extracted and summarized from 500 simulations, as shown in Table 4.

It can be seen from Figure 12 and Table 4 that the proposed method has the lowest beam entropy and the best beam convergence performance. Additionally, the beam entropy values for Cub-FDA, Rec-FDA, and Log-FDA rank second, third, and last, respectively. The results in Section 5.4 fully demonstrate that the beam formed by the proposed method has lower beam entropy, more converging beams, and better low intercept performance. The beam pooling performance reflected by beam entropy in this section is consistent with the beam diagram in Section 5.2.

(b) The method proposed in this paper is replaced by random frequency offset parameters.

**Figure 13:**
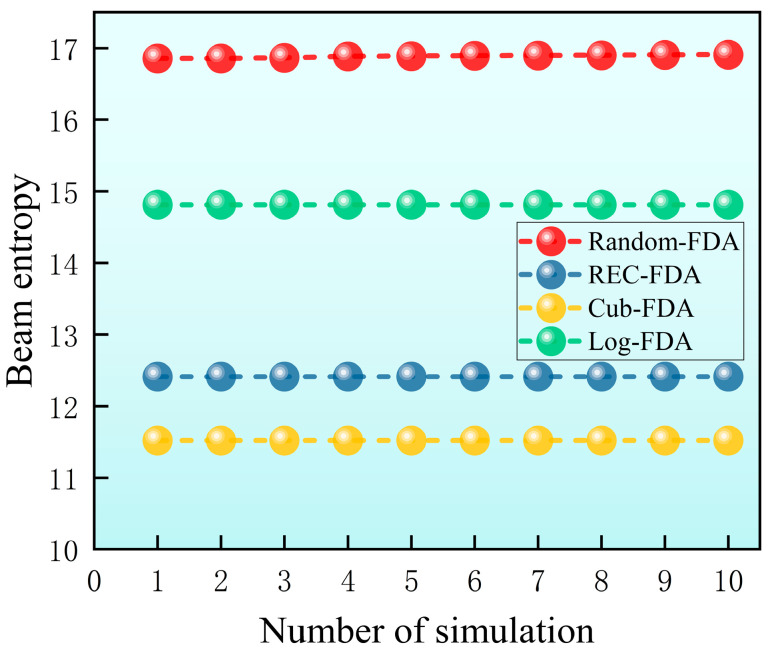
Beam entropy simulation results (in Section 5.4 (b)).

In order to compare with the simulation of Section 5.4 (a), in Section 5.4 (b), the frequency offset parameters used in the method proposed in this paper are replaced by randomly set frequency offset parameters. The frequency offset parameters of reciprocal frequency offset, exponential frequency offset, and logarithmic frequency offset are the same as those in Section 5.2, and the parameter settings are shown in Table 5. A total of 500 simulations are performed. Then, 10 minimum entropy values are selected from 500 times, and the beam entropy simulation results are drawn. The simulation results are shown in Figure 13.

As can be seen from Figure 13, the entropy of the beam formed by exponential frequency deviation is the lowest, the entropy of the beam with reciprocal frequency deviation is the second, and the entropy of the beam with random frequency deviation is the highest. When the frequency offset design method proposed in this paper is replaced by random frequency offset setting, the beam entropy increases significantly and the low intercept performance of the beam decreases significantly. This also fully shows that the frequency offset setting method proposed in this paper has important guiding significance for low intercept beam design.

### 5.5. Airspace Low Intercept Performance Analysis

**Figure 14:**
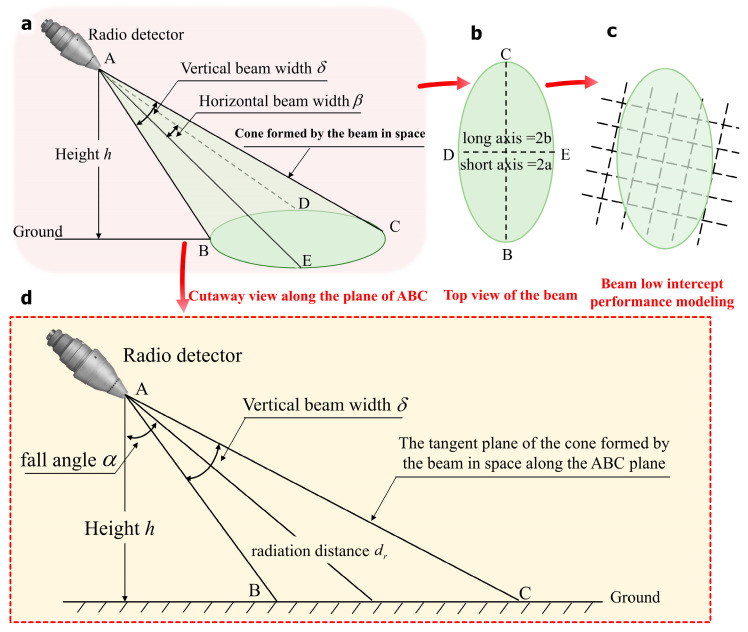
Modeling diagram of airspace low intercept performance. (**a**) Three-dimensional simulated beam pattern of radio detector; (**b**) Top view of the beam; (**c**) Beam low intercept performance modeling; (**d**) Beam 3D modeling analysis of radio detector.

Analysis of the low intercept performance of radio detector beams primarily focuses on the confrontation between the radio detector and the jammer. The RF environment model for both the radio detector and the jammer is established. Subsequently, the concept of joint intercept probability is introduced to analyze the low intercept performance of the beam. According to the above analysis, the main lobe beam of the radio detector is mainly modeled and analyzed considering the low radiation power of the radio detector. We construct the following calculation model: The beam of the radio detector forms an irregular cone in space (see Figure 14a). When the radio detector transmits detection signals to the ground, the beam intersects with the ground (horizontal plane) to form an approximate ellipse (see Figure 14b), and there are some regular grids on the ground (horizontal plane). Independent jammers (Figure 14c) are uniformly distributed above to quantify the intercept probability of the beam, and the calculation process is shown in Figure 14d. Therefore, the larger the beam width of the radio detector and the larger the approximate ellipse area results in the higher probability of interception. At the same time, it is necessary to consider the effect of interference and noise on the fuze jammer when receiving the fuze signal in the actual situation. Therefore, the derivation of airspace interception probability in this section is divided into the ideal case without noise and interference and the actual case with noise and interference.

In the process, the main parameters are as follows: ground height *h* of the radio detector, fall angle α, vertical beam width δ, horizontal beam width β, radiation distance $d_{r}$, and jammer distribution density $M_{j}$. The capital letters are used to indicate the starting and ending points of the line segment, in order to more easily describe the process of beam modeling. And the capital letters have no special meaning. If the signal power received by the jammer is greater than the minimum detectable signal power of the jammer, the probability of successful interception is *P*. Considering that each jammer is independent, the joint interception probability of all jammers in the beam coverage area to the beam can be expressed as:

(68) $$P_{total}=1-\prod_{n=1}^{N}\left(1-P_{n}\right),$$

where *N* is the number of jammers contained in the beam coverage area; $P_{n}$ is the interception probability of the *n*-th jammer. Next, the area of the approximate ellipse formed by the intersection of the beam with the ground (horizontal plane) is calculated. As can be seen from Figure 14d:

(69) $$b=\frac{1}{2}BC=\frac{1}{2}h\left[\tan\left(\alpha +\frac{\delta }{2}\right)-\tan\left(\alpha -\frac{\delta }{2}\right)\right].$$

The length of the short axis DE can be approximated as half the sum of the transverse distance corresponding to the maximum effective distance (when the apparent axis aligns with AC) and the transverse distance corresponding to the minimum longitudinal distance (when the apparent axis aligns with AB) [33]. Therefore,

(70) $$d_{\min}=AB=\frac{h}{\cos\left(\alpha -\frac{\delta }{2}\right)},$$

(71) $$d_{\max}=AC=\frac{h}{\cos\left(\alpha +\frac{\delta }{2}\right)},$$

(72) $$a=\frac{1}{2}\left(a_{\min}+a_{\max}\right)=\frac{1}{2}\left(d_{r\min}\tan\frac{\beta }{2}+d_{r\max}\tan\frac{\beta }{2}\right).$$

Thus, in the ideal case, the area of the approximate ellipse is:

(73) $$S=\pi ab=\tan\frac{\beta }{2}\cdot \frac{\pi h^{2}}{4}\left(\frac{1}{\cos\left(\alpha -\frac{\delta }{2}\right)}+\frac{1}{\cos\left(\alpha +\frac{\delta }{2}\right)}\right)\cdot \left[\tan\left(\alpha +\frac{\delta }{2}\right)-\tan\left(\alpha -\frac{\delta }{2}\right)\right].$$

In the actual case with interference and noise, the area of the approximate ellipse can be considered as the weight of the SNR of the approximate ellipse area in the ideal case. We also use Shannon weights to weight the area of the approximate ellipse. Therefore, the area of the approximate ellipse in the actual case is:

(74) $$S^{\prime }={\left(\log_{2}\left(1+\frac{S}{N}\right)\right)}^{2}\cdot \pi ab={\left(\log_{2}\left(1+\frac{S}{N}\right)\right)}^{2}\cdot \tan\frac{\beta }{2}\cdot \frac{\pi h^{2}}{4}\left(\frac{1}{\cos\left(\alpha -\frac{\theta }{2}\right)}+\frac{1}{\cos\left(\alpha +\frac{\theta }{2}\right)}\right)\cdot \left[\tan\left(\alpha +\frac{\theta }{2}\right)-\tan\left(\alpha -\frac{\theta }{2}\right)\right]$$

The following is a simulation analysis.

**Figure 15:**
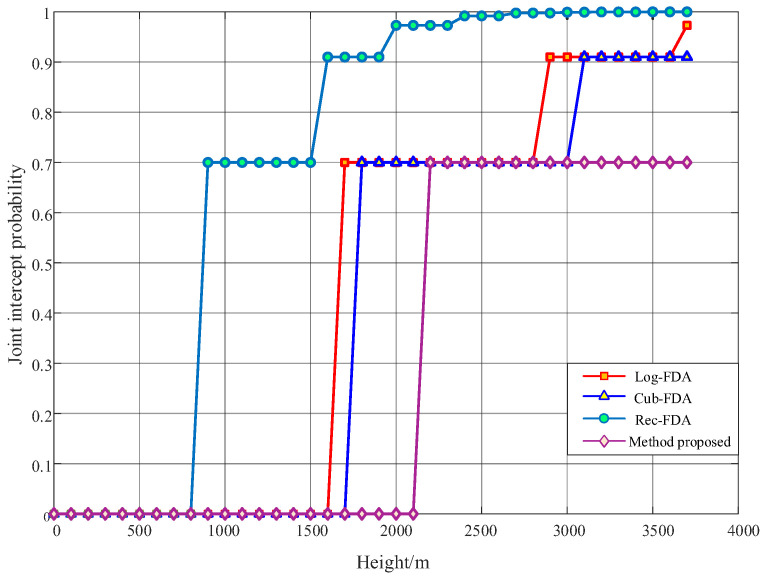
Simulation diagram of low intercept performance of radio detector beam in airspace in the ideal case.

In the simulation, we believe that the beam of the radio detector is a symmetrical pen beam, with the horizontal beam width equal to the vertical beam width (δ=β). In this section, three typical frequency offset setting methods, Log-FDA, Cub-FDA, and Rec-FDA, are used for comparison. The combined intercept probability curve of the radio detector is drawn with the altitude of the radio detector as the independent variable. The FDA-MIMO frequency offset setting parameters are listed in Table 2, while other simulation parameters of the radio detector are shown in Table 6. The simulation results are shown in Figure 15.

As illustrated in Figure 15, the joint interception probability of the Rec-FAD beam is greater than 70% when the radio detector is above 900 m from the ground. When the radio detector is positioned at an altitude exceeding 1600 m, the joint intercept probability of the beam is greater than 90%. The spatial low intercept performance of the beam is unsatisfactory, failing to meet the minimum operational distance requirement of the radio detector. The combined intercept probability of Log-FDA and exp-FDA beam is greater than 70% when the radio detector is more than 1600 m above the ground. When the detector’s altitude exceeds 3100 m, the joint interception probability surpasses 90%, which does not meet the startup distance of the radio detector, and the spatial low interception performance of the beam is second. The frequency offset setting method proposed in this paper ensures that the probability of the radio detector being intercepted within 2100 m from the ground is 0, which satisfies the starting distance of the radio detector. Throughout the entire simulation process (0–3700 m), this method achieves optimal spatial low intercept performance.

**Figure 16:**
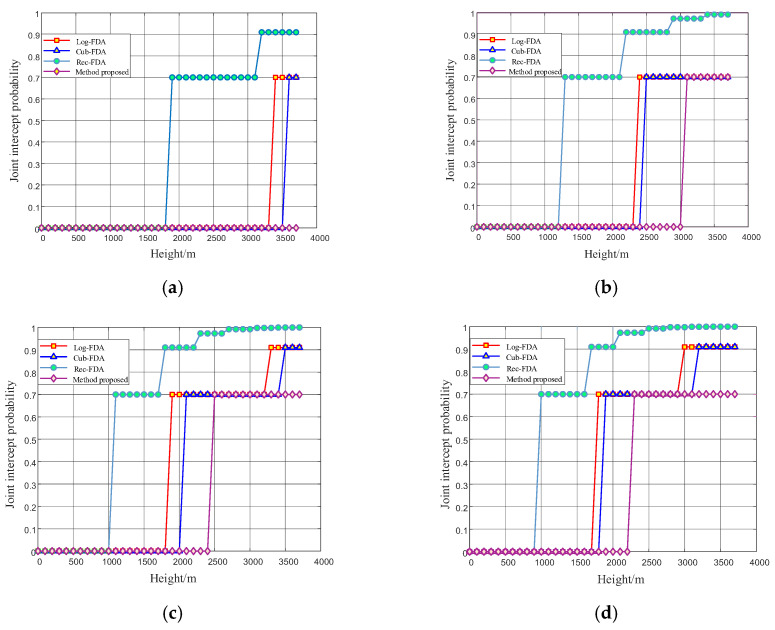
Simulation diagram of low intercept performance of radio detector beam in airspace with interference and noise. (**a**) Simulation diagram of low intercept performance of radio detector beam in airspace when the signal-to-noise ratio is −5 dB; (**b**) simulation diagram of low intercept performance of radio detector beam in airspace when the signal-to-noise ratio is 0 dB; (**c**) simulation diagram of low intercept performance of radio detector beam in airspace when the signal-to-noise ratio is 5 dB; (**d**) simulation diagram of low intercept performance of radio detector beam in airspace when the signal-to-noise ratio is 10 dB.

As can be seen from Figure 16, when there is external interference and noise, the joint interception probability of radio fuze signal at the same height is lower than the ideal situation. When the SNR is −5 dB, the interception probability of the proposed method can be 0 in the whole simulation process (0–3700 m). The spatial interception probability of the proposed algorithm is much lower than that of logarithmic frequency offset, exponential frequency offset, and reciprocal frequency offset. With the increase in SNR, the critical intercept height of the fuze proposed in this paper is always higher than the critical intercept height of logarithmic frequency offset, exponential frequency offset, and reciprocal frequency offset. When the signal to noise ratio is 10 dB, the frequency offset setting method proposed in this paper makes the probability of the fuze being intercepted within 2200 m from the ground as 0, which satisfies the starting distance of the radio fuze. It fully shows that the method proposed in this paper has the best performance in the real situation of noise and interference.

## 6. Conclusions

In this paper, the concept of beam entropy is proposed to guide the design of low intercept beams for radio detectors. Based on FDA-MIMO technology, the design principle of low interception optimal converging beam for radio detectors is presented. By strategically positioning the wave crest and power drop points within a relatively close range (Δr), the beam amplitude in a small neighborhood with a radius of Δr near the wave crest point is large, while the beam amplitude in other ranges is rapidly decreased. Furthermore, the beam function is used to calculate the frequency offset of each array element, leading to the development of a design principle centered on low interception point-like beams through this frequency offset setting method. By plotting the beam pattern, the point-like beam designed in this paper achieves a distance of 1 m and a beam width of 9 degrees. The performance of beam convergence is superior to the existing classical methods. The simulation of low interception performance based on beam entropy and spatial domain shows that the entropy of the designed beam reaches its minimum value. Additionally, the probability that the beam designed in this paper is intercepted within 2100 m from the ground is 0, which aligns well with the activation distance requirements of radio fuzes. In contrast, beams designed using existing classical methods exhibit higher entropy and inferior performance in low airspace acquisition. In summary, through rigorous formula derivation and beam simulation, the correctness of the design principle of the low interception point-like beam is verified. It provides theoretical support for the low intercept beam design of radio detectors based on FDA-MIMO. The optimal beam convergence method proposed in this paper can enhance the low intercept performance of the radio detector but also improve its anti-jamming capabilities. Moreover, the application of low interception point-beam optimal convergence design technology holds significant potential. The technology proposed in this paper has broad applications in radio fuze detection and low intercept directional communication for unmanned systems, including unmanned aerial vehicles and unmanned boats. However, the method proposed in this paper still faces the problems of a complex frequency offset calculation formula and large amounts of calculations. Therefore, in order to be widely used in practical scenarios as soon as possible, the frequency offset calculation formula should be further optimized to achieve a simple and fast calculation of point-like beam frequency offset.

## Figures and Tables

**Table 1:** Simulation parameter settings.

Parameter	Value	Parameter	Value
element number	10	point beam expected angle/°	45
center frequency/GHz	10	point beam expected distance/m	50
each array element frequency offset/MHz	0, −50.80, −51.10, −51.34, −51.64, −52.42, −52.83, −53.17, −53.27, −54.66		

**Table 2:** The frequency setting of each FDA array in simulation.

Parameter	Value	Parameter	Value
element number	10	$\mathrm{center}\;\mathrm{frequency}\;f_{0}$/GHz	3
each element frequency offset (Log)/Hz	0, 3466, 5493, 6931, 8047, 8959, 9729, 10,397, 10,986, 11,513		
each element frequency offset (Cub)/MHz	0.005, 0.04, 0.135, 0.32, 0.63, 1.08, 1.72, 2.56 3.65, 5		
each element frequency offset (Rec)/MHz	5, 2.5 1.7, 1.25, 1, 0.83, 0.7, 0.63, 0.56, 0.5		
each element frequency offset (proposed)/MHz	0, 45.7, 46.1, 46.3, 47.2, 47.5, 47.7, 47.8, 48.0, 48.2		

**Table 3:** Point-like beam performance comparison.

Method	PSR	HPBW_r_/m	HPBW_θ_/°
Log-FDA	0.54	$1.62\times 10^{4}$	11.8
Cub-FDA	0.58	33	11
Rec-FDA	0.87	$3.87\times 10^{4}$	21.4
Proposed method	0.41	1	9

**Table 4:** The optimal beam entropy in different simulation situations.

Method	Log-FDA	Cub-FDA	Rec-FDA	Method Proposed
Beam entropy	14.81	11.52	12.41	10.85

**Table 5:** Simulation parameter setting table in Section 5.4 (b).

Parameter	Value	Parameter	Value
element number	10	$\mathrm{center}\;\mathrm{frequency}\;f_{0}$/GHz	3
each element frequency offset (Log)/Hz	0, 3466, 5493, 6931, 8047, 8959, 9729, 10,397, 10,986, 11,513		
each element frequency offset (Cub)/MHz	0.005, 0.04, 0.135, 0.32, 0.63, 1.08, 1.72, 2.56 3.65, 5		
each element frequency offset (Rec)/MHz	5, 2.5 1.7, 1.25, 1, 0.83, 0.7, 0.63, 0.56, 0.5		
each element frequency offset (random)/MHz	9.1, 9.5, 5.7, 9.6, 8.2, 5.5, 6.4, 7.7, 9.8, 6.2		

**Table 6:** Airspace low intercept performance simulation parameter setting.

Parameter	Value	Beam Width	Value
fall angle/°	15	Log-FDA	11.8°
height/m	100–3700	Cub-FDA	11°
jammer distribution density/$m^{-2}$	0.0005	Rec-FDA	21.4°
intercept probability of a jammer	0.7	proposed	9°
SINR	−5, 0, 5, 10		

## Data Availability

The data presented in this study are available on request from the corresponding author.

## Abbreviations

The following abbreviations are used in this manuscript:

LPI	Low probability of intercept.
FDA	Frequency diverse array.
MIMO	Multiple-input multiple-output.
*r*	$\mathrm{The}\;\mathrm{beam}\;\mathrm{detection}\;\mathrm{distance}\;\mathrm{at}\;\mathrm{any}\;\mathrm{point}\;\left(r,\theta \right)$ within the beam detection range.
θ	$\mathrm{The}\;\mathrm{beam}\;\mathrm{detection}\;\mathrm{angle}\;\mathrm{at}\;\mathrm{any}\;\mathrm{point}\;\left(r,\theta \right)$ within the beam detection range.
$A\left(r,\theta \right)$	The amplitude of a beam at a certain position B within the beam detection range.
$f_{0}$	The center frequency of each coherent signal.
$f_{m}$	The signal frequency of the *m*-th array element.
$r_{m}$	The distance from the beam pointing position to the *m*-th array.
$r_{0}$	The distance from the beam pointing position to the first array element (reference array element).
$\Delta f_{m}$	The frequency offset of the *m*-th array relative to the reference array.
*m*	Array number.
d	Array distance.

## Appendix A

The derivation process of the beam amplitude formula when the frequency deviation increment of each array element of FDA-MIMO is evenly distributed.

The derivation of Formula (14).

Formula (13) is rewritten as follows:

(A1) $$A\left(t;r,\theta \right)=\exp\left[-j2\pi \left(f_{0}t-\frac{r_{0}}{c}f_{0}\right)\right]\;\cdot \;\sum_{m=1}^{M}\exp\left[-j2\pi \left(m-1\right)\left(\Delta ft-\frac{r_{0}}{c}\Delta f+\frac{d\sin\theta }{c}f_{0}\right)\right]$$

Due to the current main consideration of mold length, so

(A2) $$\begin{array}{ll} \left|A\left(t;r,\theta \right)\right| & =\left|\exp\left[-j2\pi \left(f_{0}t-\frac{r_{0}}{c}f_{0}\right)\right]\right|\;\cdot \;\left|\sum_{m=1}^{M}\exp\left[-j2\pi \left(m-1\right)\left(\Delta ft-\frac{r_{0}}{c}\Delta f+\frac{d\sin\theta }{c}f_{0}\right)\right]\right|\\ & =\left|\sum_{m=1}^{M}\exp\left[-j2\pi \left(m-1\right)\left(\Delta ft-\frac{r_{0}}{c}\Delta f+\frac{d\sin\theta }{c}f_{0}\right)\right]\right| \end{array}$$

where $\sum_{m=1}^{M}\exp\left[-j2\pi \left(m-1\right)\left(\Delta ft-\frac{r_{0}}{c}\Delta f+\frac{d\sin\theta }{c}f_{0}\right)\right]$ is equivalent to a geometric sequence. To facilitate the derivation of the formula, let $B=-j2\pi \left(m-1\right)\left(\Delta ft-\frac{r_{0}}{c}\Delta f+\frac{d\sin\theta }{c}f_{0}\right)$.

According to the arithmetic series sum formula $S_{n}=\frac{a_{1}\left(1-q^{n}\right)}{1-q}(q\;\mathrm{is}\;\mathrm{the}\;\mathrm{common}\;\mathrm{ratio}\;\mathrm{of}\;\mathrm{the}\;\mathrm{geometric}\;\mathrm{series})$, therefore

(A3) $$S_{n}=\sum_{m=1}^{M}\exp\left[B\right]=\frac{1-{\left(\exp\left[B\right]\right)}^{M}}{1-\exp\left[B\right]}=\frac{1-\exp\left[MB\right]}{1-\exp\left[B\right]}=\frac{1-{\left(\exp\left[\frac{MB}{2}\right]\right)}^{2}}{1-{\left(\exp\left[\frac{B}{2}\right]\right)}^{2}}$$

Take $1-{\left(\exp\left[\frac{B}{2}\right]\right)}^{2}$ as an example to further derive:

(A4) $$1-{\left(\exp\left[\frac{B}{2}\right]\right)}^{2}=1-{\left(\cos\frac{B}{2}+j\sin\frac{B}{2}\right)}^{2}=2\sin^{2}\frac{B}{2}-2j\sin\frac{B}{2}\cos\frac{B}{2}=2\sin\frac{B}{2}\left(\sin\frac{B}{2}-j\cos\frac{B}{2}\right)$$

Therefore, the Formula (A3) can be rewritten as:

(A5) $$S_{n}=\frac{2\sin\frac{MB}{2}\left(\sin\frac{MB}{2}-j\cos\frac{MB}{2}\right)}{2\sin\frac{B}{2}\left(\sin\frac{B}{2}-j\cos\frac{B}{2}\right)}=\frac{2\sin\frac{MB}{2}}{2\sin\frac{B}{2}}\;\cdot \;\frac{\sin\frac{MB}{2}-j\cos\frac{MB}{2}}{\sin\frac{B}{2}-j\cos\frac{B}{2}}$$

Formula (A2) is rewritten as:

(A6) $$\left|A\left(t;r,\theta \right)\right|=\left|\frac{2\sin\frac{MB}{2}}{2\sin\frac{B}{2}}\;\cdot \;\frac{\sin\frac{MB}{2}-j\cos\frac{MB}{2}}{\sin\frac{B}{2}-j\cos\frac{B}{2}}\right|=\left|\frac{2\sin\frac{MB}{2}}{2\sin\frac{B}{2}}\right|\cdot \left|\frac{\sin\frac{MB}{2}-j\cos\frac{MB}{2}}{\sin\frac{B}{2}-j\cos\frac{B}{2}}\right|=\frac{\sin\frac{MB}{2}}{\sin\frac{B}{2}}$$

Namely

(A7) $$\left|A\left(t;r,\theta \right)\right|=\frac{\sin\left[M\pi \left(\Delta ft-\frac{r_{0}}{c}\Delta f+\frac{d\sin\theta }{c}f_{0}\right)\right]}{\sin\left[\pi \left(\Delta ft-\frac{r_{0}}{c}\Delta f+\frac{d\sin\theta }{c}f_{0}\right)\right]}$$

## Appendix B

**Proof** **of** **Inference** **1.** When the frequency offset of each array element increases uniformly, it can be seen from the beam function (15) that each S-shaped beam has periodicity in the distance dimension. The two adjacent S-shaped beams change periodically in the distance dimension. The period is:

(A8) $$R_{z}=\frac{c}{\Delta f},$$

where *c* is the speed of light; Δf is the frequency offset of FDA-MIMO. Because the frequency offset of each array element is uniformly increased, a periodic beam pattern is generated. If a point-like beam is generated, the periodicity of the beam is destroyed, causing multiple beams to converge to form a point beam. ① The periodicity of the damaged beam requires that the frequency offset of each array element does not increase evenly. ② From the perspective of the distance period of the beam, the convergence of multiple beams to form a point-like beam effectively reduces the distance period among the beams. As can be seen from Equation (A8), an increased frequency offset for each array element facilitates a reduction in the distance period, thereby promoting the formation of a point-like beam.□

## Appendix C

**Proof** **of** **Inference** **2.** In the proof of point-beam forming, analyzing the beam amplitude of points *H* and $H^{\prime }$ separately under different frequency offset settings does not provide meaningful insights. We should focus on the ratio between the peak *H* and the power drop point $H^{\prime }$ beam amplitude to better evaluate the convergence performance of the beam. Therefore, when the frequency offset of each array element of FDA-MIMO is set in full accordance with Equation (48), the ratio $\Psi_{1}$ of wave peak point *H* and power drop point $H^{\prime }$ beam amplitude is:

(A9) $$\Psi_{1}=\frac{M^{2}+\frac{4\pi^{2}}{c^{2}}{\left[\frac{M\left(M-1\right)}{2}df_{0}\sin\theta -r_{0}\sum_{m=2}^{M}\Delta f_{m}\right]}^{2}}{M^{2}+\frac{4\pi^{2}}{c^{2}}{\left[\frac{M\left(M-1\right)}{2}df_{0}\sin\theta -r_{0}\sum_{m=2}^{M}\Delta f_{m}-M\Delta rf_{0}\right]}^{2}}.$$

When the frequency offset of each array element floats δ, the ratio $\Psi_{2}$ of the beam amplitude of the peak point *H* and the power drop point $H^{\prime }$ is:

(A10) $$\Psi_{2}=\frac{M^{2}+\frac{4\pi^{2}}{c^{2}}{\left[\frac{M\left(M-1\right)}{2}df_{0}\sin\theta -r_{0}\sum_{m=2}^{M}\left(1+\delta \right)\Delta f_{m}\right]}^{2}}{M^{2}+\frac{4\pi^{2}}{c^{2}}{\left[\frac{M\left(M-1\right)}{2}df_{0}\sin\theta -r_{0}\sum_{m=2}^{M}\left(1+\delta \right)\Delta f_{m}-M\Delta rf_{0}\right]}^{2}},$$

(A11) $$\Psi_{1}-\Psi_{2}=\frac{M^{2}+\frac{4\pi^{2}}{c^{2}}{\left[\frac{M\left(M-1\right)}{2}df_{0}\sin\theta -r_{0}\sum_{m=2}^{M}\Delta f_{m}\right]}^{2}}{M^{2}+\frac{4\pi^{2}}{c^{2}}{\left[\frac{M\left(M-1\right)}{2}df_{0}\sin\theta -r_{0}\sum_{m=2}^{M}\Delta f_{m}-M\Delta rf_{0}\right]}^{2}}-\frac{M^{2}+\frac{4\pi^{2}}{c^{2}}{\left[\frac{M\left(M-1\right)}{2}df_{0}\sin\theta -r_{0}\sum_{m=2}^{M}\left(1+\delta \right)\Delta f_{m}\right]}^{2}}{M^{2}+\frac{4\pi^{2}}{c^{2}}{\left[\frac{M\left(M-1\right)}{2}df_{0}\sin\theta -r_{0}\sum_{m=2}^{M}\left(1+\delta \right)\Delta f_{m}-M\Delta rf_{0}\right]}^{2}}.$$

Equation (A11) is simplified and approximated to:

(A12) $$\Psi_{1}-\Psi_{2}=\frac{2M^{2}r_{0}\Delta rf_{0}\delta }{\left\{M^{2}+\frac{4\pi^{2}}{c^{2}}{\left[\frac{M\left(M-1\right)}{2}df_{0}\sin\theta -r_{0}\sum_{m=2}^{M}\Delta f_{m}-M\Delta rf_{0}\right]}^{2}\right\}\cdot \left\{M^{2}+\frac{4\pi^{2}}{c^{2}}{\left[\frac{M\left(M-1\right)}{2}df_{0}\sin\theta -r_{0}\sum_{m=2}^{M}\left(1+\delta \right)\Delta f_{m}-M\Delta rf_{0}\right]}^{2}\right\}}.$$

Because $\frac{4\pi^{2}r_{0}\sum_{m=2}^{M}\Delta f_{m}}{c^{2}}\to 0$, Equation (A12) can be simplified as:

(A13) $$\Psi_{1}-\Psi_{2}=\frac{2M^{2}r_{0}\Delta rf_{0}\delta }{16\pi^{4}{\left(\frac{M\left(M-1\right)}{2}d\sin\theta -M\Delta r\right)}^{4}\frac{f_{0}^{4}}{c^{4}}}.$$

In order to ensure that the dot beam can still be formed after the frequency offset of each array element is floating, the smaller $\Psi_{1}-\Psi_{2}$ is required. Therefore, considering that the operating frequency of the radio detector is tens of GHz, when the frequency offset floating range is within 10%, the numerator and denominator of Equation (A13) are roughly the same order of magnitude, and the $\Psi_{1}-\Psi_{2}$ value is small. Therefore, when the frequency offset of each array element fluctuates within 10%, the formation of a point-like beam remains feasible.□
